# The lateral superior olive in the mouse: Two systems of projecting neurons

**DOI:** 10.3389/fncir.2022.1038500

**Published:** 2022-10-20

**Authors:** Isabella R. Williams, Anastasia Filimontseva, Catherine J. Connelly, David K. Ryugo

**Affiliations:** ^1^Garvan Institute of Medical Research, Darlinghurst, NSW, Australia; ^2^School of Medical Sciences, University of New South Wales, Kensington, NSW, Australia; ^3^Department of Otolaryngology-Head, Neck and Skull Base Surgery, St. Vincent’s Hospital, Darlinghurst, NSW, Australia

**Keywords:** anatomy, auditory, isofrequency, lateral superior olive, olivocochlear efferents, tonotopy, topography

## Abstract

The lateral superior olive (LSO) is a key structure in the central auditory system of mammals that exerts efferent control on cochlear sensitivity and is involved in the processing of binaural level differences for sound localization. Understanding how the LSO contributes to these processes requires knowledge about the resident cells and their connections with other auditory structures. We used standard histological stains and retrograde tracer injections into the inferior colliculus (IC) and cochlea in order to characterize two basic groups of neurons: (1) Principal and periolivary (PO) neurons have projections to the IC as part of the ascending auditory pathway; and (2) lateral olivocochlear (LOC) intrinsic and shell efferents have descending projections to the cochlea. Principal and intrinsic neurons are intermixed within the LSO, exhibit fusiform somata, and have disk-shaped dendritic arborizations. The principal neurons have bilateral, symmetric, and tonotopic projections to the IC. The intrinsic efferents have strictly ipsilateral projections, known to be tonotopic from previous publications. PO and shell neurons represent much smaller populations (<10% of principal and intrinsic neurons, respectively), have multipolar somata, reside outside the LSO, and have non-topographic, bilateral projections. PO and shell neurons appear to have widespread projections to their targets that imply a more diffuse modulatory function. The somata and dendrites of principal and intrinsic neurons form a laminar matrix within the LSO and share quantifiably similar alignment to the tonotopic axis. Their restricted projections emphasize the importance of frequency in binaural processing and efferent control for auditory perception. This study addressed and expanded on previous findings of cell types, circuit laterality, and projection tonotopy in the LSO of the mouse.

## Introduction

The perception of auditory space is initiated by the complementary actions of multiple auditory brainstem nuclei. Anatomical and physiological data implicate the medial superior olive in the processing of interaural time differences, the lateral superior olive (LSO) and medial nucleus of the trapezoid body (MNTB) in decoding interaural level differences, and the dorsal cochlear nucleus (CN) for analyzing spectral cues created by head and pinna reflections (Young et al., 1992; Joris, 1996; Joris and Yin, 1998; Konishi et al., 2003; Reiss and Young, 2005; Middlebrooks, 2015; Franken et al., 2018; Joris and van der Heijden, 2019). The LSO is part of the superior olivary complex (SOC), located at the base of the pontine-medullary junction and is one of the earliest structures to receive binaural inputs. Basic knowledge about LSO cell morphology and how they are synaptically connected represents an important first step to understanding the circuits for the localization and separation of sounds.

Auditory information that reaches the LSO originates directly from ipsilateral spherical bushy and planar multipolar cells of the anteroventral cochlear nucleus (AVCN; Cant and Casseday, 1986; Doucet and Ryugo, 2003) and indirectly from contralateral globular bushy cells of the AVCN by way of the MNTB (Warr, 1966; Tolbert et al., 1982; Glendenning et al., 1985; Banks and Smith, 1992; Henkel and Gabriele, 1999). The convergence of these two inputs, one excitatory and the other inhibitory, are matched in frequency tuning and temporal characteristics for analysis of interaural level differences, a primary cue for localizing high frequency sounds (Yin et al., 2019). The result of this binaural processing is sent along ascending pathways for further encoding of spatial location (Tollin, 2003; Middlebrooks and Waters, 2020) and down descending pathways to modulate cochlear sensitivity in ways yet to be fully understood.

The LSO contains a heterogeneous population of neurons that have been categorized on the basis of somatic size, dendritic morphology, location (Ollo and Schwartz, 1979; Cant, 1984; Helfert and Schwartz, 1986, 1987; Rietzel and Friauf, 1998; Kulesza, 2008), chemical markers (Helfert et al., 1989; Eybalin, 1993), or projections (Adams, 1979; Glendenning and Masterton, 1983; Henkel and Brunso-Bechtold, 1993). These different neuron groups are presumably involved in separate aspects of processing and dispensing this information. The dominant LSO cell type is the principal neuron and they have ascending projections up the midbrain (Adams, 1979; Glendenning and Masterton, 1983; Cant, 1984; Helfert and Schwartz, 1986). Other cell types have been described and vary with respect to species, staining technique, taxonomic criteria, and observer. Four cell types are proposed for the cat (Helfert and Schwartz, 1986), four for the gerbil (Helfert and Schwartz, 1987), three for the mouse (Ollo and Schwartz, 1979), three for the human (Kulesza, 2007), and seven for the rat (Rietzel and Friauf, 1998). The main limitation to these taxonomic schemes is that they do not include circuit information and they are founded on observations collected from different species of widely different ages, crucial variables known to influence structure and function (Ryugo and Fekete, 1982; Romand, 1983; Brugge, 1988; Jenkins and Simmons, 2006; Van Hooser, 2007).

The other main type of LSO neuron in the mouse is the intrinsic neuron, which comprises the group of lateral olivocochlear (LOC) efferents, whose axonal projections terminate under inner hair cells of the cochlea primarily against the peripheral processes of auditory nerve fibers (Warr and Guinan, 1979). Intrinsic neurons are intermixed with the principal cells in rodents (White and Warr, 1983; Aschoff and Ostwald, 1987; Campbell and Henson, 1988; Vetter and Mugnaini, 1992; Yao and Godfrey, 1995; Safieddine et al., 1997; Sánchez-González et al., 2003; Mulders and Robertson, 2004; Brown, 2011; Romero and Trussell, 2021). In other mammals, such as cats, squirrel monkeys, and humans, members of the LOC system may be found outside the LSO in various periolivary nuclei (Warr, 1975; Thompson and Thompson, 1986; Aschoff and Ostwald, 1987; Bishop and Henson, 1987; Brown et al., 1988; Moore, 2000). The functional significance of the location of these efferent cell bodies is unknown.

The principal neuron is the dominant cell type in the LSO but there are disagreements with respect to the laterality of their ascending projections. They have been qualitatively reported as bilateral and symmetric (Adams, 1979; Schweizer, 1981; Nordeen et al., 1983; Ross et al., 1988; Grothe, 1994; Kelly et al., 1998) or with a contralateral preference (Brunso-Bechtold et al., 1981; Willard and Martin, 1984; Moore et al., 1995; Mellott et al., 2021). There are reports that (1) ipsilateral projections are glycinergic and entirely inhibitory (Saint Marie et al., 1989; Saint Marie and Baker, 1990), (2) ipsilateral projections are primarily low frequency, whereas high frequency projections are mostly contralateral (cats, Glendenning and Masterton, 1983; Oliver, 2000); and (3) low frequencies project contralaterally, whereas high frequencies project ipsilaterally (ferrets, Henkel and Brunso-Bechtold, 1993).

A tonotopic organization has been shown for the LSO (Boudreau and Tsuchitani, 1970; Guinan et al., 1972; Sanes and Rubel, 1988; Sanes et al., 1990) and IC (Rose et al., 1963; Merzenich and Reid, 1974; FitzPatrick, 1975; Stiebler and Ehret, 1985). This organization for the LSO appears dependent on its topographic and tonotopic input from the CN as well as from the MNTB (Friauf and Ostwald, 1988; Sommer et al., 1993; Doucet and Ryugo, 2003; Gómez-Álvarez and Saldaña, 2015). The IC gets tonotopic input from the CN (Ross et al., 1988; Oliver et al., 1997; Malmierca et al., 2005). Topographic and tonotopic connections between the IC and LSO have been reported using large injections of a retrograde tracer in cats (Brunso-Bechtold et al., 1981) and rats (Kelly et al., 1998) but a more detailed analysis of this pathway is merited.

The data on the LSO have been collected under a variety of different conditions, perhaps accounting for some of the disagreements in the literature. The aims of this study in the adult mouse were (1) to address the cell types of the LSO; (2) to determine quantitatively if the projection of principal neurons to the IC is symmetric; and (3) to expand on previous findings of LSO topography and tonotopy.

## Materials and methods

### Mouse model of hearing

This study was performed in strict accordance with the Australian Code for the Care and Use of Animals for Scientific Purposes (2013). All of the animals were handled according to Animal Ethics Committee protocols (Animal Research Authority: 19-33, 20-02, and 21-13) approved by the Garvan/St Vincent’s Hospital Animal Ethics Committee. All procedures were conducted under appropriate anesthesia and analgesia with animal welfare consideration underpinned by the principles of Replacement, Reduction and Refinement. A total of 44 healthy CBA/CaH mice of either sex and between the ages of 3–8 months were used.

### Hearing status

All animals underwent auditory brainstem response testing prior to experimentation. Animals were positioned in a double-walled, sound-attenuating chamber (Sonora Technology, Gotenba, Japan) on a heating pad under ketamine/xylazine (100 mg/kg; 20 mg/kg) anesthesia. When areflexic to a toe-pinch, the recording, reference, and ground electrodes were positioned beneath the skin above the vertex, left pinna, and biceps femoris, respectively. A speaker was positioned 45° off the midline and 10 cm from the pinna where alternating condensation and rarefaction click stimuli (100 μsec square wave pulses) and tone stimuli at 4, 8, 16, 24, 32, 40, and 48 kHz (5 ms duration, 0.5 ms rise/fall) were generated using a software-controlled signal processor [RZ6/BioSigRZ; Tucker-Davis Technologies (TDT)] and delivered from 90 to 30 dB SPL in 10 dB decremental steps to either ear separately. Stimulus presentations (*n* = 512) were delivered at a rate of 10/s for each level and the evoked responses were amplified (RA16PA/RA4LI; TDT), bandpass filtered from 0.5 to 3 kHz, recorded, and averaged (RZ6; TDT). Only mice with normal auditory brainstem response thresholds and audiograms (Zheng et al., 1999; Taberner and Liberman, 2005; Muniak et al., 2018) were used in this study.

### Neuronal tract tracing

#### Inferior colliculus injections

Injections of retrograde tracers, Fluorescein Dextran (FD-3000MW, biotinylated, 5% in saline; Cat #D3305, Invitrogen/Molecular Probes, Scoresby, VIC, Australia), Fluorogold (FG; 4% in saline, Fluorochrome, Denver, CO, USA), Cholera Toxin Subunit-B (CTB; 0.5% in saline; List Biological Laboratories, Campbell, CA, USA) and Antonia Red Dextran 4 (AR; 10% saline; cat# 79672, Sigma Aldrich, St Louis, MO, USA) were made iontophoretically (Supplementary material 1) or *via* pressure (up to 0.5 μL) into the central nucleus of the IC. Pressure injections were used in the IC to maximize the labeling of the principal neurons in the LSO, particularly to show their neuronal distribution within the nucleus.

The IC surgical approach began by making a skin incision on the dorsal surface of the head to expose the cranial sutures, bregma and lambda. Approximately 5.2 mm posterior to bregma, a craniotomy (roughly 2 mm^2^) was made overlying the IC. A glass micropipette (20–60 μm, inside tip diameter) was positioned on a micromanipulator and used to pressure inject 0.5 μL of tracer (100 nL/min) into the IC at a depth of 1.0–1.5 mm (stereotaxic coordinates of Paxinos and Franklin, 2008). Bilateral injections into each IC were performed with FD and FG to visualize the bilateral projection property of both IC (as above, *n* = 7). Following IC injections, bone wax was applied to cover the craniotomy, and VetBond tissue adhesive was used to close the incision site for the post-surgical survival period. Retrograde tracers were placed in both the IC and cochlea of a mouse (*n* = 4) in order to label LSO neurons with ascending and descending projections in the same LSO.

#### Cochlea injections

The surgical approach to the cochlea involved a post-auricular incision and removal of the tympanic membrane and the ossicular chain. With the middle ear opened, a microliter syringe was used to inject 0.5–1 μL of tracer directly into the round window (*n* = 21). After injection, the round window was plugged with bone-wax to prevent tracer leakage, bupivacaine (0.05 mL) was injected subcutaneously at the incision site, and VetBond was used to close the incision. The animal survived 14 days following the injection.

### Tissue processing

Animals were euthanized with an intraperitoneal injection of Lethabarb (0.1 mg/kg) and perfused transcardially with 3–5 mL of 1% sodium nitrate in phosphate-buffered saline, followed by 60 mL of 4% paraformaldehyde (0.1M phosphate buffer, pH 7.4). The head was removed, the calvaria partially opened to expose the brain, and the head post-fixed for two-three hours. The brain was then completely dissected out of the skull and post-fixed overnight at room temperature in 0.1M buffered 4% paraformaldehyde. The next day, the brain was embedded in a gelatin-albumin mixture hardened with 4% paraformaldehyde and sectioned into 60 μm-thick sections using a vibrating microtome (Leica VT1200S, Nussloch, DE).

Cresyl violet (CV) staining was routinely performed on sections mounted on slides using a protocol modified from Humason (1979). The sections were hydrated in distilled water for 5 min, followed by a 10-min incubation in 0.1% CV dye at room temperature. The slides were rinsed in distilled water, followed by rinses in 70% alcohol, 95% alcohol and then differentiated (95% alcohol with 10 drops of glacial acetic acid) for one minute to remove excess staining. Rehydration in decreasing concentration of alcohol (one-minute periods in 70, 50, 30%, and distilled water) further removes excess CV for air-drying overnight and cover slipping with Permount the next day.

### Cholinergic staining

Cholinergic markers, choline acetyltransferase (ChAT; *n* = 7) or acetylcholinesterase (AChE; *n* = 3), were used to visualize the cholinergic neurons of the LSO. The two methods were used to confirm cell counts and size measurements. Immunohistochemical processing of ChAT was performed on free-floating sections. Sections were washed 3x for 5 min each in 0.12M-Tris buffered saline, placed in 3% hydrogen peroxide for 10 min, followed by washes with Tris buffered saline, incubated in 0.1% Photoflo (Kodak, Rochester, NY, USA) for 1 h, and followed by an hour in 10% normal goat serum. Sections were washed and incubated at 4°C overnight in 1:1000 mouse anti-ChAT primary antibody (Cat #VP- C3838; RRID:AB_2336337; Vector Labs, Newark, CA, USA) and 2% normal goat serum. Negative control sections were unexposed to primary antibody. The following day, sections were rinsed and incubated for one hour in 1:200 biotinylated goat anti-mouse secondary antibody (Cat #BA-9200, Vector Labs, Burlingame, CA, USA). Sections were rinsed and then developed in a solution of 0.005% 3,3’-diaminobenzene (DAB) with 0.03% hydrogen peroxide until a distinct brown reaction product appeared in the tissue or intensified by the addition of nickel ammonium sulfate to produce a deep purple reaction product (NiDAB). All sections were mounted and coverslipped with Permount (ThermoFisher, Waltham, MA, USA) for examination by brightfield microscopy.

Cholinergic staining for AChE was performed on glass-mounted tissue sections using a standard protocol (Karnovsky and Roots, 1964). Briefly, the slides were incubated in acetylcholine medium for 30 minutes, followed by 6 × 30 s rinses in distilled water, then incubated in 4% sodium sulfide solution for 1 min, followed by 2 × 30 s rinses in distilled water. The tissue was “toned” in 1% silver nitrate for 30 s, rinsed 6 × 30 s in distilled water, air dried overnight, and then coverslipped with Permount. LOC cell counts and measurements confirmed labeling equality for ChAT and AChE.

### Fluorescent tracer processing

All cases with fluorescent tracer injections were visualized with standard fluorescent microscopy. The tissue sections were cut, mounted immediately, and coverslipped with VectaShield (H-1400; Vector Labs, Newark, CA, USA). In cases where chromogenic processing was performed after fluorescent imaging, the coverslips were removed, and the tissue was processed accordingly.

### Immunohistochemical processing of neuronal tracers

Thirteen injection cases with tracer deposits of FG, FD, and/or CTB were processed by chromogenic development. Free-floating tissue sections were placed in serial order in 24-well plates. Sections were washed in 0.12M Tris buffered saline, treated with 3% hydrogen peroxide for 10 min, rinsed, and permeabilized in 0.1% Photoflo (Kodak, Rochester, NY, USA) for one hour. Tissue processed for biotinylated FD were then incubated for one hour in avidin-biotin complex (Vectastain Elite ABC Kit, Cat# PK-6100; Vector Labs, Newark, CA, USA) before undergoing development with DAB (as above).

Tissue processed for FG were incubated for one hour in 10% normal goat serum (Cat#VES100020, Vector Labs, Newark, CA, USA), whereas CTB-tissue was placed in 1% normal rabbit serum (Cat # S-5000; Vector Laboratories, Burlingame, CA, USA). The tissue then underwent 3x rinses for 5 min, before being placed at 4°C overnight in 1:100 rabbit anti-FG primary antibody and 2% normal rabbit serum (Cat#R4880, Sigma Aldrich, St Louis, MO, USA) or polyclonal goat anti-CTB primary antibody (1:10,000; Cat# 703, RRID:AB_10013220; List Biological Laboratories) with 2% normal goat serum. Negative control sections were not exposed to primary antibody. FG sections were rinsed and incubated in biotinylated goat anti-rabbit secondary (Cat #AB207995, Abcam, Cambridge, United Kingdom) and CTB sections were incubated in biotinylated rabbit anti-goat secondary (1:200; Cat# BA-5000; Vector Labs) for one hour, rinsed, and incubated for one hour in avidin-biotin complex. Sections were developed with either 0.005% 3,3’-diaminobenzene (DAB) or nickel-intensified DAB (NiDAB). Some cases were counterstained with CV. In cases where two tracers were injected, FD was processed prior to FG. All sections were mounted and coverslipped with Permount for examination with brightfield microscopy.

### Quantification of the lateral superior olive neuronal cohorts and statistics

Photomontages of serial sections were created from low magnifications (2.5x, 10x, and 20x objectives) from the facial nucleus to the ventral nucleus of the lateral lemniscus to identify the boundary of the LSO. Cell measurements of the different neuron types was made from high magnification brightfield and fluorescent micrographs (40x Plan-Apochromat or 100x Neofluar objectives). Micrographs of the LSO was created by making z-stacks from 4 to 5 focal planes using *Photoshop* software (300 dpi resolution) (Adobe Photoshop, 2021), and the micrographs were pieced together into montages to cover the entire LSO. Only cells with a visible nucleus were included for analysis. The size of principal and intrinsic neurons were compared in male and female mice aged from 3 to 8 months and the sizes of principal cells, POs, intrinsic cells, and shell neurons were all compared with respect to the different staining methods. There is no statistical size difference created by age, sex, or staining methods for the data reported (Supplementary material 2 and Supplementary Tables 1–3). Positive controls for our cell size method were provided by results calculated from vesicular Glutamate Transporter-2-stained principal cells and AChE-stained intrinsic cells of the Allen Brain Atlas (Brain Map - brain-map.org, 2021). There was no difference when comparing average somatic areas from our tissue to these other two data sets (vesicular Glutamate Transporter-2: *p* = 0.2009; AChE: *p* = 0.3118, *Welch’s t-test*).

The ratio of ipsilateral and contralateral projecting principal neurons was calculated from bilateral counts of projecting principal neurons from the most rostral to the most caudal LSO sections. The cell body had to be contained inside the fiber-lined border LSO border (principal and intrinsic neurons) or within approximately 100 μm of the LSO boundary (POs and shells). Cell position was plotted onto the outline of the LSO to create maps of cell projection patterns. Somatic silhouette area was calculated using *FIJI* software (Schindelin et al., 2012).

The methods for evaluating somatic and dendritic alignment and for confirming cellular alignment to the tonotopic axis are described in Supplementary materials 3, 4, respectively. The cell body silhouette area, neuronal counts, angle difference between somatic long axis and dendritic vector, and orientation of LSO neurons to the tonotopic axis were subject to *Descriptive Statistics*, *Welch’s t-test, and Two-way ANOVA* using *Šídák’s Multiple Comparison Test* ((Prism 9, GraphPad Software (2021), San Diego, CA, USA). *Note*: Statistical analyses that compare two cohorts tested for significance in *Prism* Software using the *Welch’s t-test*; statical analyses comparing more than two groups were tested for significance using a *Two-way ANOVA* test. Means and standard deviations, *p-values*, and statistical tests are provided.

## Results

The goal of this study was to begin a systematic description of some LSO circuits in the mouse in the context of various conflicting reports on the nucleus made in different mammalian species. We used a range of frequency-defined injection sites to describe the tonotopic relationship between principal cells of the LSO and the IC, applied quantitative methods to determine the laterality of these projections, and re-visited LSO cell categories with a focus on LOC efferents.

### Labeling of principal and periolivary lateral superior olive neurons

**FIGURE 1:**
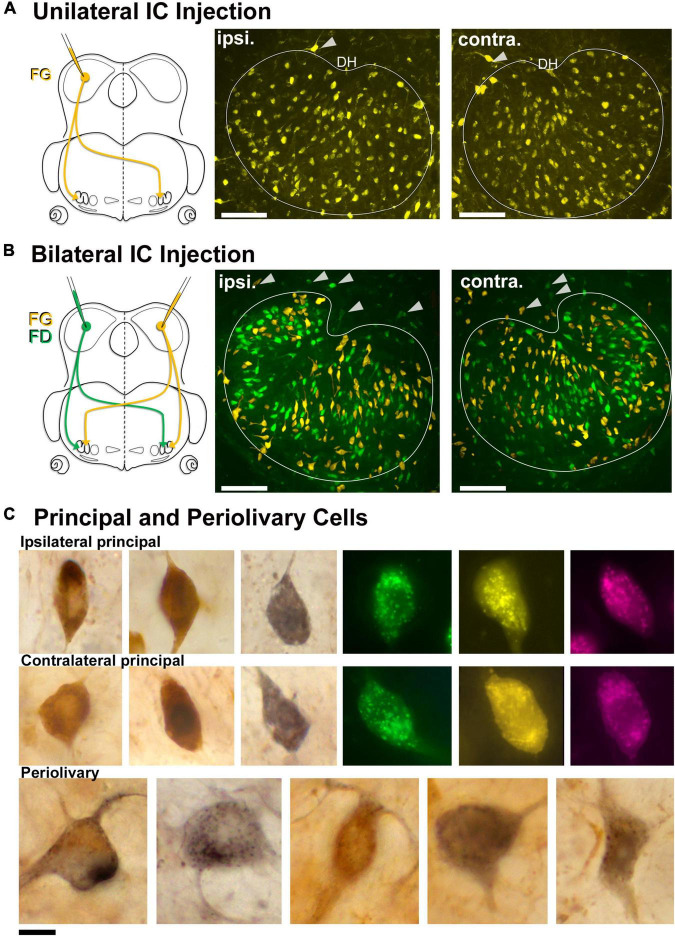
Retrogradely labeled LSO principal neurons from unilateral and bilateral tracer injections into the CNIC. **(A)** A unilateral injection of a retrograde tracer into the CNIC resulted in bilateral labeling of LSO principal neurons. Schematic representation of FluoroGold (FG) injected into the CNIC to label the neurons in the LSO with ascending projections. Photomicrographs (20x objective) of the ipsilateral and contralateral LSO showing FG-labeled principal neurons. *Gray arrowheads:* PO neurons on the borders of the LSO. **(B)** Schematic illustration of the retrograde tracers FG (yellow) and FD (green) injected into the right and left IC, respectively (same animal), to label the principal neurons in the LSO. Photomicrographs (20x objective) of the ipsilateral and contralateral LSO showing FG and FD labeled principal neurons in the same nucleus. *Gray arrowheads:* PO neurons on the borders of the LSO. **(C)** Photomicrographs (100x objective) of the ipsilateral (top row) and contralateral (middle row) principal cells labeled from chromogenic development of FG with DAB (brown) or NiDAB (black) and fluorescent tracers (FD-green, FG-yellow, Antionia Red-magenta). The principal neurons were fusiform with unipolar or bipolar dendritic extensions. The preolivary neurons were also labeled (bottom row) and featured a large, polygonal cell body using chromogenic development. ipsi., ipsilateral; contra., contralateral; FG, fluorogold; FD, fluorescein dextran. Scale bar equals 100 μm **(A,B)**, 25 μm **(C)**.

Unilateral injections of the retrograde tracers, FD, FG, and/or AR, were made into the CNIC to label LSO neurons with ascending projections (Figure 1). Initially, pressure injections were made to get a global view of the connections, where unilateral injections labeled cells in both the ipsilateral and contralateral LSO (Figure 1A). The labeled neurons were distributed uniformly and appeared homogenous in appearance, except for some neurons on the border of the nucleus. The main neurons exhibited a general orientation toward the dorsal hilus (DH) and fit the descriptions of LSO principal neurons (Ollo and Schwartz, 1979; Cant, 1984). A small number of topographically labeled neurons were located on the border of the nucleus and often conformed to the border’s shape; these matched the description of marginal cells (Ollo and Schwartz, 1979; Rietzel and Friauf, 1998). Cells with a larger cell body and dendrites that did not exhibit any particular orientation were occasionally labeled and found outside the LSO proper (Figure 1A, arrowheads). These were the PO neurons of the LSO as described in the cat (Adams, 1979, 1980).

Bilateral injections into the left and right CNIC labeled ipsilateral and contralateral LSO neurons side-by-side throughout the nucleus and were indistinguishable except by tracer (Figure 1B). Principal cells had elongated cell bodies of generally equal size (ipsilateral, 129.3 ± 37.37 μm^2^; contralateral 131.2 ± 36.87 μm^2^, Supplementary Table 3) with a marked orientation toward the DH. No neurons were double labeled, indicating that a single neuron did not give rise to an ascending axon that innervated both ICs. A much smaller number (<25) of large, multipolar PO cells (198.5 ± 30.2 μm^2^, Table 1) were labeled by all IC injections, and these were scattered around the outside of the LSO (Figures 1A,B). A comparison of ipsilateral and contralateral principal cell body size and shape can be made and considered with respect to that of POs (Figure 1C).

**TABLE 1:** Cell body silhouette area (μm2) for the principal and periolivary (PO) neurons and the intrinsic and shell efferent neurons.

Neurons	Principal	PO	Intrinsic	Shell	Large CV
Number of cases	3	3	4	4	2
Number of cells	439	41	261	33	166
Median (μ m^2^)	117.9	199.8	96.61	155.2	107.7
Mean (μ m^2^) ± Standard deviation	123.9 ± 26.56	198.5 ± 30.17	97.33 ± 26.27	161.1 ± 28.26	111.8 ± 37.00

Two-way testing was used to compare the soma silhouette area of all subtypes of LSO neurons assessed in this study. A two-way ANOVA showed significant differences existed between the principal, PO, intrinsic, and shell neurons, but no significant difference occurred between the CV labeled neurons and the principal neurons [*F*(105,438) = 0.86, *p* = 0.8408] or the CV labeled neurons and the intrinsic efferents [*F*(1,103) = 3.09, *p* = 0.0816].

### Labeling of lateral olivocochlear efferents

**FIGURE 2:**
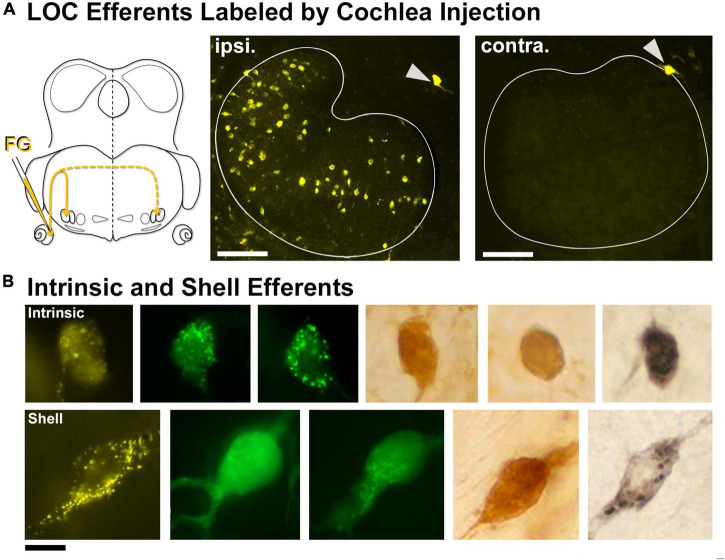
Labeled lateral olivocochlear (LOC) efferents following retrograde tracer injections into the cochlea. **(A)** Schematic illustration of retrograde tracer injected through the round window of the left cochlea. Solid yellow line indicates the primary pathway to the ipsilateral LOCs, whereas the dashed line leads to the very few contralateral LOCs. Gray arrowheads: shell neurons on the borders of the LSO. **(B)** Photomicrograph (100x objective) of the intrinsic LOC efferents (top row) labeled with chromogenic development (DAB-brown, NiDAB-black) and with fluorescent markers (FG-yellow, FD-green). The intrinsic neurons were small and round, and look similar to the principal neurons. The shell neurons (bottom row) were labeled in the same tissue as the intrinsic neurons, and featured a large cell body with broader dendritic extensions. ipsi., ipsilateral; contra., contralateral; FG, fluorogold; FD, fluorescein dextran. Scale bar equals 100 μm **(A)**, 25 μm **(B)**.

Lateral olivocochlear efferents were labeled by pressure injections of the retrograde tracers FD, FG, or CTB into the round window of the cochlea (Figure 2A). The labeling for intrinsic efferents, found within the LSO proper, was entirely ipsilateral. Labeled cells were oval with thin unipolar or bipolar dendritic stalks generally evident on opposite poles. The intrinsic efferents appeared to align perpendicularly to the borders of the LSO nucleus. Their somata were slightly smaller (97.33 ± 26.27 μm^2^, Table 1) than those of principal cells but their shape was roughly the same (Figures 2A,B, row 1). A few labeled shell efferents were found bilaterally and outside the LSO borders, often near the DH (Figure 2A, arrowheads). Shell neurons were larger than intrinsic neurons (average area, 161.1 ± 28.3 μm^2^, Figure 2B, bottom row and Table 1).

**FIGURE 3:**
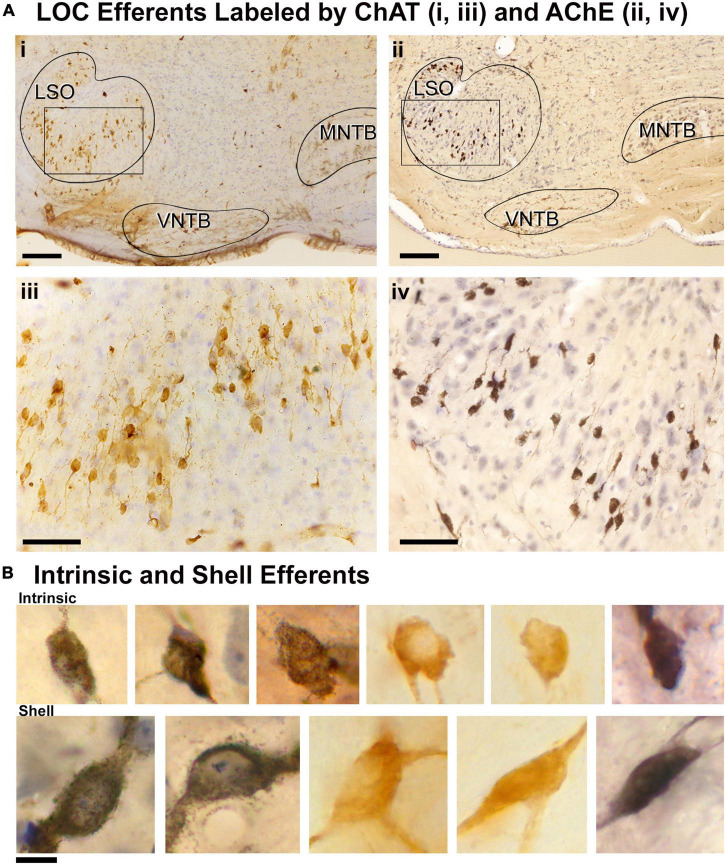
Labeled cholinergic LOC efferent neurons. LOC neurons were labeled using cholinergic markers, ChAT and AChE, and counterstained with CV. **(Ai)** Photomicrograph (10x objective) of the superior olivary complex (SOC) region labeled by ChAT immunostaining. **(ii)** Photomicrograph (10x objective) of the SOC region labeled by AChE staining. **(iii,iv)** Higher magnification micrographs (40x objective) of inset **(i,ii)**, showing the ChAT and AChE labeled LOC neurons contained within the LSO, respectively. Note the similarity of ChAT and AChE labeling. **(B)** Photomicrographs (100x objective) showing the cholinergic LOC neurons labeled from either ChAT or AChE staining. The intrinsic neurons (top row) feature fusiform somata and were distributed throughout the core of the LSO nucleus. The shell neurons (bottom row) were larger and more globular in shape. LSO, lateral superior olive; VNTB, ventral nucleus of the trapezoid body; MNTB, medial nucleus of the trapezoid body. Scale bar equals 250 μm **(Ai,ii)**, 50 μm **(Aiii,iv)**, 25 μm **(B)**.

ChAT or AChE was used to determine total efferent cell distribution (Figure 3A) as well as cell morphology (Figure 3B) because they had previously been shown to label LOC efferents in the mouse (Safieddine et al., 1997; Brown, 2011). Intrinsic cells were orientated in line with the tonotopic axis of the LSO (Figure 3A) as previously reported (Vetter and Mugnaini, 1992; Brown, 2011). ChAT and AChE staining confirmed that the two methods exhibited equal sensitivity for counting (ChAT: average total, 362.0 ± 25.41; AChE: average total, 357.8 ± 18.63; *p* = 0.7532; Table 2) and size measurements (Supplementary Table 3). Intrinsic efferents had fusiform-shaped cell bodies and the dye often extended into the primary dendrite (Figure 3B, top row). The labeling of the intrinsic and shell neurons with cholinergic staining was consistent with the LOC efferents labeled by retrograde tracers (Figure 3).

**TABLE 2:** Counts comparing the distribution of LOC efferents stained by AChE or ChAT.

Case	Stain	Count
*AM4*	AChE	343
*AM5*	AChE	344
*AM11*	AChE	389
*AM298*	AChE	331
*AM1348*	ChAT	385
*AM1351*	ChAT	380
*AM1353*	ChAT	382
*AM1417*	ChAT	376
*AM1419*	ChAT	331
*AM1450*	AChE	354
*AM1454*	AChE	355
*AM1477*	ChAT	349
*Average AChE*	357.8 ± 18.63	
*Average ChAT*	362.0 ± 25.41	
*Combined average*	359.9 ± 21.35	

The number of LOC efferent neurons were calculated to compare the labeling distribution between the two stains. The average count for AChE and average count for ChAT were not significantly different (*p* = 0.7532).

**FIGURE 4:**
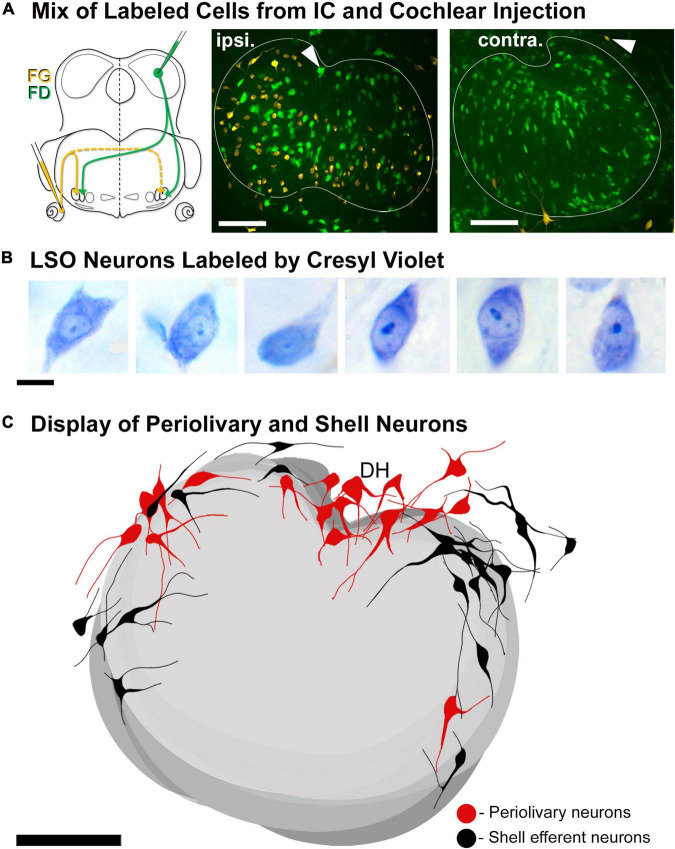
Double injection of retrograde tracers into the right IC and left cochlea to label the LSO neurons with ascending projections and LOC efferents with descending projections, respectively. LSO neurons with ascending and descending projections were labeled *via* tracer injections of FD into the right CNIC and FG into the round window of the left cochlea, respectively. **(A)** Schematic illustration of the injection sites and pathways for the projecting neurons. Fluorescent micrograph showing the left LSO containing labeled contralateral principal neurons (FD-green) and ipsilateral LOC efferents (FG-yellow). The LOC efferents were primarily ipsilaterally projecting, with only a few shell neurons projecting contralaterally. *Gray arrowheads:* PO neurons (green fluorescence) and shell neurons (yellow fluorescence) on the borders of the LSO. **(B)** Photomicrographs (100x objective) of CV labeled LSO neurons. In tissue stained by CV, we were unable to distinguish principal from intrinsic neurons due to the similarity in size and shape. **(C)** Summary of location of labeled periolivary and shell neurons around the borders of the LSO. The position of PO (red) and shell (black) neurons are shown collapsed across 18 LSO sections to illustrate their spatial distribution around the LSO. ipsi., ipsilateral; contra., contralateral; FG, fluorogold; FD, fluorescein dextran. Scale bar equals 100 μm **(A,C)**, 25 μm **(B)**.

Double labeling experiments were used to reveal principal and intrinsic neurons within the same mouse. An injection of one tracer into the CNIC and an injection of a second tracer into the cochlea on the opposite side permitted direct side-by-side comparisons of principal and intrinsic neurons (Figure 4A). Principal neurons were distributed bilaterally throughout the LSO. In contrast, the intrinsic neurons were unilateral to the cochlear injection and tended to avoid the border of the nucleus. No neuron was double labeled, indicating that no neuron gave rise to a branched axon that innervated the separate targets. When tissue was stained only by CV, it was impossible to distinguish principal from intrinsic cells (Figure 4B). The PO and shell neurons were observed scattered around outside the LSO borders and were always found in association with retrogradely labeled principal and intrinsic neurons, respectively (Figure 4C).

### Topographic connections between lateral superior olive principal cells and the central nucleus of the inferior colliculus

**FIGURE 5:**
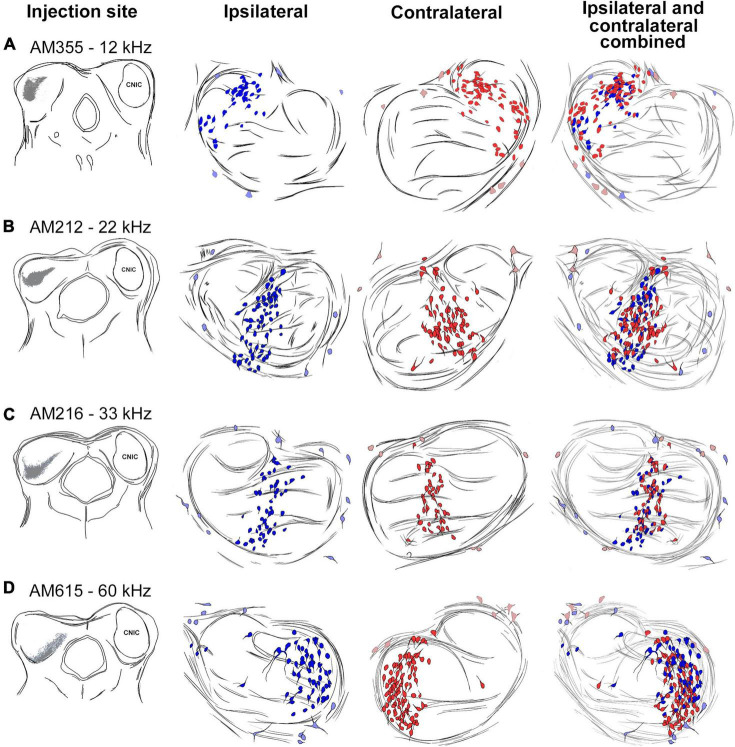
Topographic relationship between the IC and the LSO. Tracing of IC injection sites progressing more ventral and higher in frequency [**(A–D)**, left column]. The corresponding bilateral labeling of LSO principal cells (ipsilateral-dark blue, contralateral-dark red, middle two columns) and periolivary (PO) cells (ipsilateral-light blue, contralateral-light red) were traced through serial sections, aligned using blood vessels, and merged. The ipsilateral and contralateral labeling for each injection was combined (last row) to show the bilateral preservation of topographic and tonotopic organization of principal cells across the LSO. PO cells do not observe a tonotopic distribution (light blue and red). kHz, kilohertz.

Thirteen mice received iontophoretic injections of a retrograde tracer into the CNIC at an identified frequency location (Supplementary Figure 1). Four representative cases are shown to illustrate the topography and the bilateral symmetry in the projection (Figure 5). Plots from three adjacent sections were transferred onto the section representing the 50th percentile. The labeled cells occupy a relatively circumscribed region in the LSO. In terms of topography, note how the progressively deeper IC injections with higher frequencies (Figures 5, left column, 6A) create labeling of principal cells in the LSO that move e*n masse* as a “stripe” from lateral to medial (Figures 5, middle columns, 6B). There is also a scattering of labeled PO neurons found just outside the LSO, and these occur in predictably low numbers but in variable locations. The pattern of labeling was similar for all cases and independent of the retrograde tracer used, involving principal, marginal, and PO cells.

**FIGURE 6:**
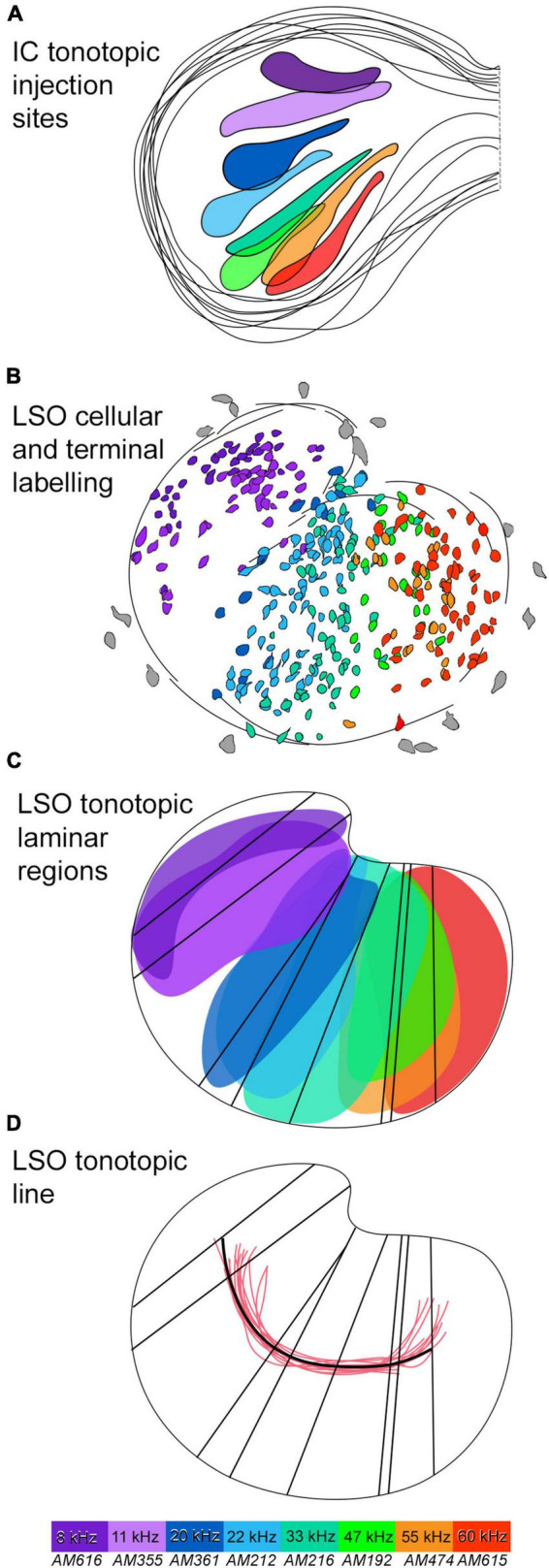
Tonotopic relationship between the IC and LSO with a tonotopic axis. **(A)** Several IC injections were made in the dorsal to ventral regions of the CNIC to labeled the correspondingly low to high frequency output in the LSO. 8 IC injection sites were drawn and superimposed to present an IC frequency representation. Frequencies included: 8, 11, 20, 22, 33, 47, 55, and 60 kHz (color coded). **(B)** The corresponding label of principal neurons in the LSO, color coded to match. The labeled principal cells show the LSO low to high frequency organization progressing from the lateral to medial limb, respectively. **(C)** The corresponding LSO neurons labeled from each IC injection were color coded. A region for each color of principal cells representing one frequency was drawn by outline the area of labeled cells to form a lamina for each frequency. A line representing the long axis of each frequency region was drawn to represent an isofrequency line. **(D)** A line (black) representing the tonotopic axis was drawn by connecting centroids of all the isofrequency lines and was compared to other tonotopic axis lines (red) derived from the Hamilton Jacobi output from other LSO sections. A color map is included, with purple corresponding to labeled elements from 8 kHz, and red corresponding to the highest frequency elements of 60 kHz. IC, inferior colliculus; LSO, lateral superior olive; kHz, kilohertz.

The bilateral symmetry of the retrograde labeling was assessed by copying the plot from right 50th percentile of the LSO, flipping the image in the horizontal plane, and then superimposing the flipped image onto the original left LSO (Figure 5, far right column). The mirror imaging of the right and left plots confirms the symmetry. The spatial balance was also evident by the equal numbers of ipsilateral and contralateral labeled cells whose ratio averaged 1.05 ± 0.16 (Table 3).

**TABLE 3:** Counts comparing the distribution between the ipsilateral and contralateral principal neurons of five cases.

Case	Tracer	Total ipsilateral cells	Total contralateral cells	Total count	Ratio
*AM1360*	FD	362	350	712	1.03
*AM1362*	FD	812	685	1497	1.18
*AM1496*	FD	273	232	505	1.17
*AM1496*	FG	370	382	752	0.969
*AM1507*	FD	203	263	466	0.772
*AM1507*	FG	743	589	1332	1.26
*AM1526*	FG	160	160	320	1

The number of ipsilateral and contralateral principal neurons were calculated to compare the labeling distribution between both sides. The ratio was computed by dividing the total ipsilateral cells of each case by the total contralateral cells. A ratio closest to 1, infers a labeling distribution that is most comparable between the two sides. The average ratio for these seven cases is 1.05 ± 0.16. Cases mentioned more than once were double labeled with FD and FG tracers.

While the “sheets” of labeled cells exhibit a tonotopic organization, there is also spatial overlap of principal cells that exhibited separate but similar frequency characteristics (Figure 6B). This overlap of labeled cells in the LSO reflects the overlap observed in the IC injection sites having different but nearby frequency characteristics (Figure 6A). The spatial spread in the distribution of labeling may be a result of combining data from different cases onto a model LSO. PO cell labeling was relatively invariant, regardless of the location of the IC injection (Figure 5, middle columns). Every part of the CNIC appears innervated by both populations of cells with principal and marginal cells having restricted projections and PO cells having widespread projections.

### Tonotopic axis

The angle of the somatic long axis compared to the corresponding angle of its dendritic vector for principal and intrinsic neurons was small, indicating alignment of these two features: (principal cells: 7.24 ± 10.42°; intrinsic efferents: 6.49 ± 9.33°; Supplementary Table 4). This result inferred that the cell body pointed in the direction of the dendritic trajectory (further details in Supplementary material 3) and allowed us to quantify the somatic orientation of LSO cells to the tonotopic axis (Supplementary material 4).

The principal cells created a columnar profile that defined an “isofrequency” sheet (Figures 5, 6B) that ran the length of the LSO, hinged near the DH. An isofrequency sheet for each case was laid out on the 50th percentile of the nucleus (Figure 6C). A centroid was calculated for each isofrequency sheet (FIJI) and black vertical lines were drawn through the centroids to create a long axis line for each sheet (Figure 6C). The centroids were connected by a black line that represented the tonotopic axis of the nucleus (Figure 6D).

The Hamilton–Jacobi Skeleton algorithm (He et al., 2021), which bisects a complex structure by following the curvature of the opposing borders, was used to create a representative tonotopic axis line for 14 LSO sections (Figure 6D, red lines). This output closely matched the tonotopic axis defined by us [Figure 6D, black line; *Welch’s test (p* = 0.2967)]. The spatial representation of different isofrequency regions appear uniformly distributed across the LSO, at least up to 60 kHz, suggesting no augmented frequency representation. A line through the 30 kHz region would essentially bisect the LSO into lateral and medial halves.

**FIGURE 7:**
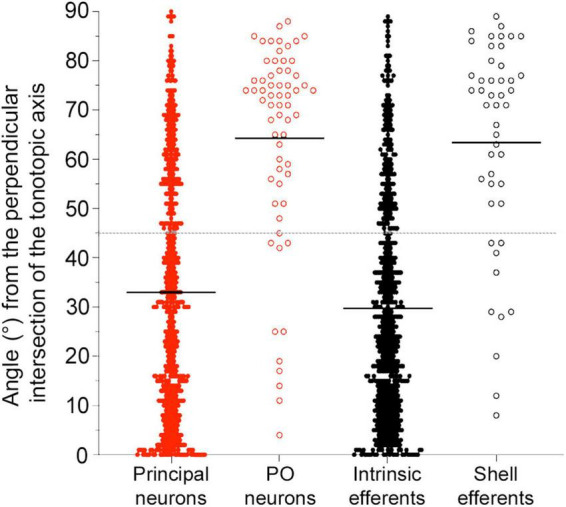
Plot of the angles for LSO neurons with ascending (red) or descending (black) projecting axons with respect to the tonotopic axis. Angle measurements for the four types of LSO neurons are shown and combined from three regions of the LSO (rostral, middle, and caudal sections). The average angle (black line) was presented for all subtypes. *Gray dashed line:* 45-degree threshold. Principal and intrinsic neurons illustrate similar angle deviations, with mean alignment below the 45-degree threshold across all planes. Periolivary (PO) neurons and shell efferent neurons both had values above the 45-degree threshold for all planes. Smaller values indicate alignment with the frequency organization. PO, periolivary.

The somatic long axes of principal and intrinsic neurons were placed onto our LSO model (Supplementary material 4) and the intersecting angle θ was measured with respect to the tonotopic axis. As suggested from the literature, LSO neurons are expected to be oriented at right angles to the tonotopic axis (Scheibel and Scheibel, 1974; Friauf et al., 2019). Each angle was reported as an absolute value and subtracted from 90°. Principal and intrinsic neurons exhibited a somato-dendritic orientation that aligned to one another and was mostly orthogonal to the tonotopic axis (Figure 7; principal neuron mean of all LSO sections = 32.99 ± 24.55°; intrinsic neuron mean = 29.70 ± 22.51°). The arrangement was not perfect but the tendency was definitely present. The PO neurons and shell efferents, however, exhibited somato-dendritic morphology that did not contribute to the structural laminae (middle, PO mean = 61.28 ± 24.62°; shell efferent mean = 59.71 ± 24.39°, Supplementary Table 5). These measurements confirmed the more orthogonal appearance of these two cell types with respect to the tonotopic axis of the LSO.

## Discussion

**FIGURE 8:**
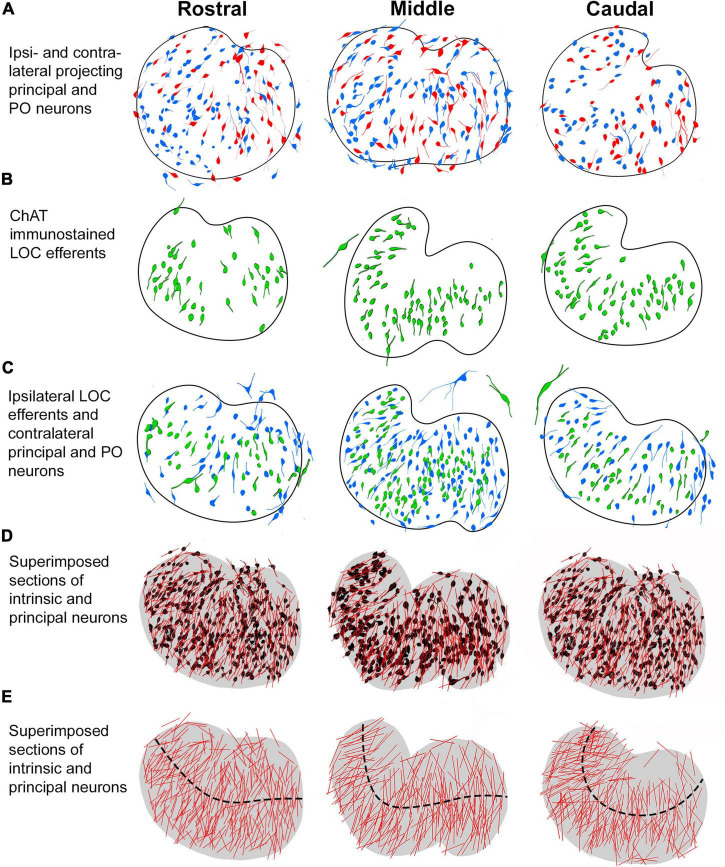
Summary of labeling pattern of principal and intrinsic neurons in rostral, middle, and caudal LSO sections. The projecting cell types were traced and mapped to illustrate their distribution in representative anterior-posterior regions of the nucleus. **(A)** Ipsilateral (red) and contralateral (blue) principal neurons labeled from bilateral CNIC injections. **(B)** Intrinsic efferent neurons labeled by ChAT immunostaining (green). **(C)** Contralateral projecting principal neurons (blue) and ipsilateral projecting intrinsic neurons (green) labeled by retrograde tracer injections into the CNIC and cochlea, respectively. The larger neurons on the borders of the LSO are identified as the PO and shell neurons [seen in **(A–C)**]. **(D)** LSO neurons with ascending or descending projections labeled from separate LSO sections were superimposed and collapsed as one color (black) on a representative rostral, middle, and caudal LSO section. The somatic long axis line (red) is contrasted against the individual neurons. **(E)** A model LSO representing rostral, middle, and caudal sections was drawn (gray, fourth row). Here, the relative alignment of principal and intrinsic neurons are shown with the representative tonotopic axis (black dashed line). An overall organization within the nucleus is seen to comprise fibrodendritic laminae. PO, periolivary; LOC, lateral olivocochlear.

The present study provides a qualitative and quantitative anatomical assessment of four types of LSO projecting neurons with connections with the IC or cochlea. Retrograde tracers placed into the CNIC labeled neurons with ascending projections (principal and PO neurons), whereas neurons with descending projections were labeled by injections of retrograde tracers deposited into the cochlea or with cholinergic markers (intrinsic and shell LOC efferents). The projection laterality of the principal neurons to the IC was determined by comparing the number of ipsilateral and contralateral labeled neurons from unilateral IC injections and found to be essentially equal. The tonotopic alignment of the four subtypes of LSO neurons was examined and quantified to develop ideas about frequency specificity and possible frequency enhancement with regard to connections between the LSO and the IC. Features of somatic morphology were established to supplement connectivity data and to help distinguish principal and intrinsic neurons. These data are summarized in Figure 8.

In the mouse, principal and intrinsic neurons are spatially intermixed and have similar somatic size, shape, and dendritic morphology. Collectively, these neurons give the LSO a laminar texture and are structurally specialized to receive restricted input, which endows each with a well-defined receptive field. These cells are typical of classic lemniscal sensory neurons (Calford et al., 1983; He, 2003; Anderson and Linden, 2011).

### Lateral superior olive principal neurons: Projection symmetry and topography

Lateral superior olive cells have been divided into separate categories based on soma-dendritic features, cell body size, and location within the LSO nucleus (Ollo and Schwartz, 1979; Cant, 1984; Rietzel and Friauf, 1998), chemical markers (Helfert et al., 1989; Eybalin, 1993) or projections (Adams, 1979; Glendenning and Masterton, 1983; Henkel and Brunso-Bechtold, 1993). There is general agreement as to the main types of neurons—principal, periolivary, intrinsic, and shell— but not for all. The previously mentioned marginal cells, with their bipolar appearance and topographic relationship with the IC, may simply be principal cells that lie on the borders of the LSO (Ollo and Schwartz, 1979; Rietzel and Friauf, 1998). Bipolar, unipolar, and banana-like cells of the rat exhibit disk-shaped dendritic trees and may also represent different subgroups of principal cells with variations in location and chemistry (Rietzel and Friauf, 1998). LSO cell taxonomy is confounded by observations drawn from different species of different ages using different methods. The resolution of cell types will ultimately depend on their physiological properties and the nature of the synaptic circuits to which they belong.

We demonstrated a strict topographic projection from the LSO to the IC, which augmented previous but less detailed reports in cats and rats (Brunso-Bechtold et al., 1981; Kelly et al., 1998). Our isofrequency layers of the IC closely corresponded to tonotopic maps where isofrequency lines were drawn by connecting points of common frequencies (Stiebler and Ehret, 1985; Portfors et al., 2011). The congruency of these isofrequency layers is remarkable given that these maps were generated years apart by different methods, using different mouse strains, exposed to surgical perturbations and electrode penetrations, and subject to tissue stress by histological processing (Supplementary material 5).

Following our frequency-guided unilateral IC injections, there are unlabeled cells among the frequency-dependent labeled cells. Some of the unlabeled cells would be principal cells projecting to the other IC and others would be intrinsic efferents. Still others could have projections to different nuclei such as the nuclei of the lateral lemniscus, AVCN and dorsal cochlear nucleus (Browner and Webster, 1975; Glendenning et al., 1981; Glendenning and Masterton, 1983). There was also more spatial overlap of cells from different frequency zones than expected when combining data from different cases onto an LSO model. These border irregularities might simply reflect the combining of data across different animals. Alternatively, they could imply that the system is inherently noisy because it reflects the naturally occurring acoustic environment. That is, we rarely, if ever, encounter pure tones. Rather, we hear complex sounds such as speech, with time-varying frequencies and amplitudes, in the presence of random noises, all occurring at once and from different sources. Could it be that sound perception and discrimination are learned probability functions rather than a precise hard-wired process like the keyboard of a piano? Maybe topographic brain maps are only approximate blueprints for brain function: the auditory world is uniquely created for each individual animal by populations of neurons that learn to work together over time. Their frequency preference is acquired by their relative position in the auditory system and refined by experience but perhaps not dictated entirely by innate and immutable frequency-specific responses.

The pattern of labeled neurons in the ipsilateral and contralateral LSO following a unilateral injection of a retrograde tracer into the mouse IC was not only topographic but also symmetric. If we assume that ipsilateral and contralateral projecting neurons have similar uptake and transport efficiency for detectability, then we could anticipate that the ratio of ipsilateral versus contralateral projecting cells, regardless of the size of the injection, ought to be stable, at least for injections contained within the same IC subdivisions. On average, an equal number of labeled principal cells were reliably observed in each LSO from a unilateral injection in the CNIC (1.05 ± 0.16). The gerbil, in spite of considerable variability, exhibited a similar mean ratio (0.94 ± 0.59, Mellott et al., 2021) consistent with qualitative conclusions made for cats (Adams, 1979), ferrets (Henkel and Brunso-Bechtold, 1993), and rats (Beyerl, 1978; Fredrich et al., 2009). Other researchers reported differences in ipsi- versus contra- projections and these could be due to species differences or methods. Part of the difference could also be that the tracers being used currently are significantly more sensitive than horseradish peroxidase, which was used in many of the older publications.

The neurons lying outside the LSO with larger somata, multipolar dendrites, and ascending projections to the IC are analogous to the PO neurons in cats (Adams, 1979, 1980) and gerbils (Schofield and Cant, 1992). POs tended to be concentrated around the DH but could be found anywhere in the vicinity of the LSO and connected to the ipsilateral or contralateral IC but not to both. Some members of this population labeled with every IC injection but their numbers were relatively low and their location unpredictable. This pattern of labeling suggested a diffuse projection to the IC and their widely branching dendrites seemed suited to intercept input from a wide swath of incoming fibers.

The POs represent what had been called isodendritic neurons commonly found in the brain stem reticular system or the intralaminar (posterior) nuclei of the thalamus (Ramon-Moliner, 1962; Morest, 1964; Scheibel and Scheibel, 1966; Lu et al., 2009). Such cells receive anatomically heterogeneous input from the spinal cord and medial lemniscus (Lund and Webster, 1967; Malmierca et al., 2011), demonstrate wide receptive field properties (Wepsic, 1966; Erickson et al., 1967; Aitkin, 1973), exhibit distinct neurochemical differences (Lu et al., 2009), and have been considered part of a multimodal pathway for integrative functions (Liu et al., 2022). Could POs be part of the non-lemniscal sensory system? Regardless, these anatomical data imply sensitivity to a range of different kinds of inputs and projections that exert a more modulatory upstream influence.

### Labeling of lateral superior olive neurons with descending projections

Intrinsic neurons of the mouse exhibit bipolar morphology that resembles that of the principal neurons. They have also been shown to have restricted terminations in the inner hair cell region of the ipsilateral cochlea (Warr et al., 1997). In contrast, shell neurons have larger cell bodies and exhibit multipolar, radiating dendrites. Importantly, evidence supports the notion that shell neurons give rise to branching axons with generally a bidirectional course along the cochlear spiral with *en passant* and terminal swellings extending a tonotopic range of 1–2 octaves (Brown, 1987, 1993; Warr et al., 1997). The diffuse and expansive projections of shell neurons are consistent with characteristic attributed to polysensory, non-lemniscal cells (Morest, 1964; Hu, 2003; Kimura et al., 2003; Komura et al., 2003; Anderson and Linden, 2011).

Long-term acquired hearing loss does not affect the size, number, or ratio of ipsilateral:contralateral projecting OC efferent neurons when comparing CBA/CaH, DBA/2 and shaker-2 (sh2/sh2) mice at 6 months of age (Suthakar, 2016). Both DBA/2 and sh2/sh2 mice exhibited ABR thresholds exceeding 100 dB SPL at this age. From these observations and the relatively short post-surgical survival of our animals, we infer that our tracer injections into the cochlea did not alter the somatic structure of auditory efferents as seen through a light microscope.

### Considerations of lateral superior olive laminar organization and tonotopy

The concept of a laminar organization of auditory structures (Rockel and Jones, 1973) fostered the idea for a topologic representation of isofrequency layers that underpinned tonotopy (Boudreau and Tsuchitani, 1970; Aitkin and Webster, 1971; Guinan et al., 1972; Merzenich and Reid, 1974; Bourk et al., 1981; Imig and Morel, 1985; Friauf and Ostwald, 1988; Banks and Smith, 1992; Ryugo and May, 1993; Sommer et al., 1993; Spirou et al., 1993; Doucet and Ryugo, 2003; Malmierca et al., 2005; Muniak et al., 2013; Gómez-Álvarez and Saldaña, 2015). Previous studies utilized qualitative observations with Golgi staining to label the extensive dendritic branches of LSO neurons and suggested a laminar organization that appeared perpendicular to the tonotopic axis (Ramón y Cajal, 1909; Scheibel and Scheibel, 1974; Cant, 1984; Helfert and Schwartz, 1987; Vetter and Mugnaini, 1992; Henkel and Brunso-Bechtold, 1998; Gómez-Álvarez and Saldaña, 2015). The utility of quantitative analyses using vectors and angles demonstrated a laminar organization of LSO neurons in the human (Kulesza, 2007) and a non-laminar organization of MOC neurons in the mouse (Brown and Levine, 2008). We extended these observations by showing principal and intrinsic neurons conform to the laminar organization of the LSO, whereas PO and shell neurons do not. The observation that MOCs are sharply tuned (Liberman and Brown, 1986) even though they are multipolar with radiating dendrites (Brown and Levine, 2008) implies that frequency-specific inputs are concentrated on the cell body or proximal dendrites, not along the entire dendritic domain.

In the LSO, principal cells contribute to the isofrequency organization and are sharply tuned (Guinan et al., 1972; Tsuchitani, 1977; Sanes and Rubel, 1988; Tollin and Yin, 2002). In the LSO of the mouse, principal and intrinsic neurons adopt a strict laminar organization. On this basis, we predict that LOC neurons will exhibit sharp frequency tuning like that of LSO principal cells, although physiological recordings have not yet been made from LOC neurons. Another enigma about LOCs is that in many species, they are located outside of the LSO (Warr, 1975; Thompson and Thompson, 1986). The predominantly ipsilateral projecting efferents in the squirrel monkey have small, round-oval somata that could represent LOCs. The elongated neurons that exhibited bilateral projections were embedded in surrounding fibers of the SOC (Thompson and Thompson, 1986); their projection pattern and fibrodendritic alignment make them candidates for the sharply tuned MOCs.

### Comparative issues of the lateral superior olive

There is no uniform agreement with respect to the neurochemistry of LSO neurons, due in part to species variations in cellular composition, cell body versus terminal staining, age of animals studied, and history of noise exposure (Helfert et al., 1989; Vetter et al., 1991; Vetter and Mugnaini, 1992; Eybalin, 1993; Kandler et al., 2002; Maison et al., 2002; Nabekura et al., 2003; Niu et al., 2004; Jenkins and Simmons, 2006; Wu et al., 2020). For the cat and guinea pig, the LSO cell population seems to be almost equally divided between excitatory and inhibitory cells (Helfert et al., 1989; Glendenning et al., 1992). In the gerbil, 75% of the LSO cells are reported to be glutamatergic (Mellott et al., 2021). The projections of LSO principal neurons to the IC have been considered a key to understanding the role of excitation and inhibition in the process of binaural interactions but there is disagreement concerning many of the very basic issues of the circuitry. Different species exhibit variable immunochemical properties of LSO neurons and variations in the laterality of projections with respect to frequency and transmitter (Glendenning and Masterton, 1983; Saint Marie et al., 1989; Saint Marie and Baker, 1990; Finlayson and Caspary, 1991; Glendenning et al., 1992; Henkel and Brunso-Bechtold, 1993; Brunso-Bechtold et al., 1994; Oliver, 2000). Projections of the LSO to the IC are not distinguishable by glycinergic or glutamatergic features alone (Fredrich et al., 2009; Mellott et al., 2021). Sorting out the details of these projections in different species will be important to understand the cellular mechanisms of binaural level processing and warrants separate studies that focus on excitation and inhibition by using pathway tracing, synaptic analyses, and markers for glycine and glutamate.

The mouse has primarily high frequency hearing, unlike that of the cat, ferret, chinchilla, gerbil, guinea pig, and human. It can therefore be predicted that there will be anatomical specializations associated with the animal’s natural history, ecological niche, hearing range, and conspecific communication requirements. In rodents, for example, the known natural habitat of the gerbil is an underground burrow (Fisher and Llewellyn, 1978); it should not be surprising if its LSO differs from rodents living above-ground. In burrows, there is reduced propagation of high frequencies (Okanoya et al., 2018) and little opportunity for detecting lateralized sounds (Heth et al., 1986; Barker et al., 2021). It is, however, curious that three burrowing rodents, the gerbil, mountain beaver, and naked mole rat, have vastly different audible hearing ranges (Merzenich et al., 1973; Heffner and Heffner, 1993; Okanoya et al., 2018). In comparison, the mouse lives in open fields and urban developments and has high frequency hearing to assist in conspecific communication and danger detection (Ehret and Riecke, 2001; Portfors and Perkel, 2014). Our results provide new quantitative details on the auditory anatomy of the mouse but emphasize the importance of comparative studies if we are to better our understanding of mammalian hearing.

## Data availability statement

The original contributions presented in this study are included in the article/Supplementary material, further inquiries can be directed to the corresponding author.

## Ethics statement

This study was reviewed and approved by the Garvan/St Vincent’s Hospital Animal Ethics Committee. This study was performed in strict accordance with the Australian Code for the Care and Use of Animals for Scientific Purposes (2013) and the ethical guidelines of the National Health and Medical Research Council (NHMRC) of Australia. All animals were handled according to Animal Ethics Committee protocols (Animal Research Authority: 19-33, 20-02, and 21-13).

## Author contributions

IRW, AF, and DKR designed the research and created the figures. IRW, AF, and CJC conducted animal work and histological processing. IRW and AF analyzed the data under the supervision of DKR. IRW and DKR composed the first draft. DKR secured funding for the project. All authors contributed to the final draft of the manuscript and verify the accuracy and integrity of the work.

## Abbreviations

AChE: acetylcholinesterase
AR: antonia red dextran 4
AVCN: anteroventral cochlear nucleus
C: caudal
CF: characteristic frequency
ChAT: choline acetyltransferase
CN: cochlear nucleus
CNIC: central nucleus of the inferior colliculus
CTB: cholera toxin subunit-B
CV: cresyl violet
D: dorsal
DH: dorsal hilus
DAB: 3,3’-diaminobenzene
FD: fluorescein dextran
FG: fluorogold
IC: inferior colliculus
L: lateral
LOC: lateral olivocochlear
LSO: lateral superior olive
MNTB: medial nucleus of the trapezoid body
MOC: medial olivocochlear
NiDAB: nickel intensified DAB
OC: olivocochlear
PO: periolivary
SOC: superior olivary complex.
