# Corrigendum: The lateral superior olive in the mouse: Two systems of projecting neurons

**DOI:** 10.3389/fncir.2022.1123350

**Published:** 2023-01-04

**Authors:** Isabella R. Williams, Anastasia Filimontseva, Catherine J. Connelly, David K. Ryugo

**Affiliations:** ^1^Garvan Institute of Medical Research, Darlinghurst, NSW, Australia; ^2^School of Medical Sciences, University of New South Wales, Kensington, NSW, Australia; ^3^Department of Otolaryngology-Head, Neck and Skull Base Surgery, St. Vincent's Hospital, Darlinghurst, NSW, Australia

**Keywords:** anatomy, auditory, isofrequency, lateral superior olive, olivocochlear efferents, tonotopy, topography

In the published article, there was an error in the legend for Figures: **Figure 1, Figure 2, Figure 3, Figure 4, Figure 5, Figure 6, Figure 7, Figure 8, Supplementary Figure 5**. All figures contain the statement below that should have only been included for **Supplementary Figure 5**:

“The image approval for our adaptation of Stiebler and Ehret (1985) from John Wiley and Sons *via* the Copyright Clearance Centre; license number 5402831110296.”

The corrected legends appear below:

**Figure 1**. Retrogradely labeled LSO principal neurons from unilateral and bilateral tracer injections into the CNIC. **(A)** A unilateral injection of a retrograde tracer into the CNIC resulted in bilateral labeling of LSO principal neurons. Schematic representation of FluoroGold (FG) injected into the CNIC to label the neurons in the LSO with ascending projections. Photomicrographs (20x objective) of the ipsilateral and contralateral LSO showing FG-labeled principal neurons. Gray arrowheads: PO neurons on the borders of the LSO. **(B)** Schematic illustration of the retrograde tracers FG (yellow) and FD (green) injected into the right and left IC, respectively (same animal), to label the principal neurons in the LSO. Photomicrographs (20x objective) of the ipsilateral and contralateral LSO showing FG and FD labeled principal neurons in the same nucleus. *Gray arrowheads*: PO neurons on the borders of the LSO. **(C)** Photomicrographs (100x objective) of the ipsilateral (top row) and contralateral (middle row) principal cells labeled from chromogenic development of FG with DAB (brown) or NiDAB (black) and fluorescent tracers (FD-green, FG-yellow, Antionia Red-magenta). The principal neurons were fusiform with unipolar or bipolar dendritic extensions. The preolivary neurons were also labeled (bottom row) and featured a large, polygonal cell body using chromogenic development. ipsi., ipsilateral; contra., contralateral; FG, fluorogold; FD, fluorescein dextran. Scale bar equals 100 μm **(A,B)**, 25 μm **(C)**.

**Figure 2**. Labeled lateral olivocochlear (LOC) efferents following retrograde tracer injections into the cochlea. **(A)** Schematic illustration of retrograde tracer injected through the round window of the left cochlea. Solid yellow line indicates the primary pathway to the ipsilateral LOCs, whereas the dashed line leads to the very few contralateral LOCs. Gray arrowheads: shell neurons on the borders of the LSO. **(B)** Photomicrograph (100x objective) of the intrinsic LOC efferents (top row) labeled with chromogenic development (DAB-brown, NiDAB-black) and with fluorescent markers (FG-yellow, FD-green). The intrinsic neurons were small and round, and look similar to the principal neurons. The shell neurons (bottom row) were labeled in the same tissue as the intrinsic neurons, and featured a large cell body with broader dendritic extensions. ipsi., ipsilateral; contra., contralateral; FG, fluorogold; FD, fluorescein dextran. Scale bar equals 100 μm **(A)**, 25 μm **(B)**.

**Figure 3**. Labeled cholinergic LOC efferent neurons. LOC neurons were labeled using cholinergic markers, ChAT and AChE, and counterstained with CV. **(Ai)** Photomicrograph (10x objective) of the superior olivary complex (SOC) region labeled by ChAT immunostaining. **(ii)** Photomicrograph (10x objective) of the SOC region labeled by AChE staining. **(iii,iv)** Higher magnification micrographs (40x objective) of inset **(i,ii)**, showing the ChAT and AChE labeled LOC neurons contained within the LSO, respectively. Note the similarity of ChAT and AChE labeling. **(B)** Photomicrographs (100x objective) showing the cholinergic LOC neurons labeled from either ChAT or AChE staining. The intrinsic neurons (top row) feature fusiform somata and were distributed throughout the core of the LSO nucleus. The shell neurons (bottom row) were larger and more globular in shape. LSO, lateral superior olive; VNTB, ventral nucleus of the trapezoid body; MNTB, medial nucleus of the trapezoid body. Scale bar equals 250 μm **(Ai,ii)**, 50 μm **(Aiii,iv)**, 25 μm **(B)**.

**Figure 4**. Double injection of retrograde tracers into the right IC and left cochlea to label the LSO neurons with ascending projections and LOC efferents with descending projections, respectively. LSO neurons with ascending and descending projections were labeled *via* tracer injections of FD into the right CNIC and FG into the round window of the left cochlea, respectively. **(A)** Schematic illustration of the injection sites and pathways for the projecting neurons. Fluorescent micrograph showing the left LSO containing labeled contralateral principal neurons (FD-green) and ipsilateral LOC efferents (FG-yellow). The LOC efferents were primarily ipsilaterally projecting, with only a few shell neurons projecting contralaterally. *Gray arrowheads:* PO neurons (green fluorescence) and shell neurons (yellow fluorescence) on the borders of the LSO. **(B)** Photomicrographs (100x objective) of CV labeled LSO neurons. In tissue stained by CV, we were unable to distinguish principal from intrinsic neurons due to the similarity in size and shape. **(C)** Summary of location of labeled periolivary and shell neurons around the borders of the LSO. The position of PO (red) and shell (black) neurons are shown collapsed across 18 LSO sections to illustrate their spatial distribution around the LSO. ipsi., ipsilateral; contra., contralateral; FG, fluorogold; FD, fluorescein dextran. Scale bar equals 100 μm **(A,C)**, 25 μm **(B)**.

**Figure 5**. Topographic relationship between the IC and the LSO. Tracing of IC injection sites progressing more ventral and higher in frequency [**(A–D)**, left column]. The corresponding bilateral labeling of LSO principal cells (ipsilateral-dark blue, contralateral-dark red, middle two columns) and periolivary (PO) cells (ipsilateral-light blue, contralateral-light red) were traced through serial sections, aligned using blood vessels, and merged. The ipsilateral and contralateral labeling for each injection was combined (last row) to show the bilateral preservation of topographic and tonotopic organization of principal cells across the LSO. PO cells do not observe a tonotopic distribution (light blue and red). kHz, kilohertz.

**Figure 6**. Tonotopic relationship between the IC and LSO with a tonotopic axis. **(A)** Several IC injections were made in the dorsal to ventral regions of the CNIC to labeled the correspondingly low to high frequency output in the LSO. 8 IC injection sites were drawn and superimposed to present an IC frequency representation. Frequencies included: 8, 11, 20, 22, 33, 47, 55, and 60 kHz (color coded). **(B)** The corresponding label of principal neurons in the LSO, color coded to match. The labeled principal cells show the LSO low to high frequency organization progressing from the lateral to medial limb, respectively. **(C)** The corresponding LSO neurons labeled from each IC injection were color coded. A region for each color of principal cells representing one frequency was drawn by outline the area of labeled cells to form a lamina for each frequency. A line representing the long axis of each frequency region was drawn to represent an isofrequency line. **(D)** A line (black) representing the tonotopic axis was drawn by connecting centroids of all the isofrequency lines and was compared to other tonotopic axis lines (red) derived from the Hamilton Jacobi output from other LSO sections. A color map is included, with purple corresponding to labeled elements from 8 kHz, and red corresponding to the highest frequency elements of 60 kHz. IC, inferior colliculus; LSO, lateral superior olive; kHz, kilohertz.

**Figure 7**. Plot of the angles for LSO neurons with ascending (red) or descending (black) projecting axons with respect to the tonotopic axis. Angle measurements for the four types of LSO neurons are shown and combined from three regions of the LSO (rostral, middle, and caudal sections). The average angle (black line) was presented for all subtypes. *Gray dashed line:* 45-degree threshold. Principal and intrinsic neurons illustrate similar angle deviations, with mean alignment below the 45-degree threshold across all planes. Periolivary (PO) neurons and shell efferent neurons both had values above the 45-degree threshold for all planes. Smaller values indicate alignment with the frequency organization. PO, periolivary.

**Figure 8**. Summary of labeling pattern of principal and intrinsic neurons in rostral, middle, and caudal LSO sections. The projecting cell types were traced and mapped to illustrate their distribution in representative anterior-posterior regions of the nucleus. **(A)** Ipsilateral (red) and contralateral (blue) principal neurons labeled from bilateral CNIC injections. **(B)** Intrinsic efferent neurons labeled by ChAT immunostaining (green). **(C)** Contralateral projecting principal neurons (blue) and ipsilateral projecting intrinsic neurons (green) labeled by retrograde tracer injections into the CNIC and cochlea, respectively. The larger neurons on the borders of the LSO are identified as the PO and shell neurons [seen in **(A–C)**]. **(D)** LSO neurons with ascending or descending projections labeled from separate LSO sections were superimposed and collapsed as one color (black) on a representative rostral, middle, and caudal LSO section. The somatic long axis line (red) is contrasted against the individual neurons. **(E)** A model LSO representing rostral, middle, and caudal sections was drawn (gray, fourth row). Here, the relative alignment of principal and intrinsic neurons are shown with the representative tonotopic axis (black dashed line). An overall organization within the nucleus is seen to comprise fibrodendritic laminae. PO, periolivary; LOC, lateral olivocochlear.

**Supplementary Figure 5**. Comparison of anatomically derived isofrequency layers (left) and electrophysiologically derived isofrequency lines (right). The anatomical laminae represent a collection of reconstructed profiles of chromogenically-recovered injection sites at their midpoint from CBA/CaH mice. Profiles are color coded with respect to the frequency spectrum (lower left). The isofrequency lines were taken from **Figure 7B** (Stiebler and Ehret, 1985) where they connected points of equivalent frequency as recorded in the IC of the house mouse. We color coded their map to the frequency spectrum. The tonotopic organization for the IC is remarkably consistent given the different methods, histology, mouse strains, and eras. kHz, kilohertz. The image approval for our adaptation of Stiebler and Ehret (1985) from John Wiley and Sons *via* the Copyright Clearance Centre; license number 5402831110296.

The authors apologize for this error and state that this does not change the scientific conclusions of the article in any way. The original article has been updated.

## References

[B1] Stiebler I. Ehret G. (1985). Inferior colliculus of the house mouse. I. A quantitative study of tonotopic organization, frequency representation, and tone-threshold distribution. J. Comp. Neurol. 238, 65–76. 10.1002/cne.902380106 4044904

