# Supplementary material for: The lateral superior olive in the mouse: Two systems of projecting neurons
Source: Front Neural Circuits. 2022 Oct 20;16:1038500. doi: 10.3389/fncir.2022.1038500 (PMC9630946; doi:10.3389/fncir.2022.1038500)
Supplement: Supplementary file 1 [file Data_Sheet_1.pdf]

Supplementary Material:

Supplementary Material 1 – Electrophysiology and IC Injections

Animals were anesthetized (ketamine/xylazine, 100mg/kg; 20mg/kg), secured in a stereotaxic frame (David Kopf Instruments, Tujunga, CA, USA), and positioned in an electrically shielded, sound-attenuated chamber (Sonora Technology Co., Yokohama, Japan) on a heating pad. Bone wax covering the craniotomy was removed just before recording. Quartz glass micropipette electrodes were used for multi-unit recordings (inner tip diameter 15-20μm). They were filled with a solution of 0.15M Tris Buffer, pH 7.3, 3M KCl, and a retrograde tracer: Fluorescein Dextran (5% in saline); Fluorogold (4% in saline,), Cholera Toxin Subunit-B (0.5% in saline), or Antonia Red Dextran 4 (10% saline).

Stimulus delivery and neural recordings were controlled via custom software. Acoustic stimuli were generated digitally (DAP5016a; Microstar Laboratories, Bellevue, WA, USA), amplified (Halo A23; Parasound, San Francisco, CA, USA), attenuated (PA5; Tucker Davis Technologies, Alachua, FL, USA), and delivered by a calibrated free-field speaker (EMIT High Energy; Infinity, La Crescent, MN, USA) placed 10 cm from the mouse and 45° off the midline into the sound field contralateral to the structure under investigation. Search stimuli were 200 msec broadband noise bursts or sinusoidal pure tones (4/sec) delivered as the recording electrode was advanced into the brain using a motorized hydraulic micromanipulator (2650; David Kopf Instruments). Characteristic frequency (CF), the frequency to which a cell is most sensitive, and threshold of the CF were determined audiovisually while manually adjusting the tone burst frequency and attenuation. CF was then confirmed with an automated tuning curve protocol that measured responses to a 4-octave (oct.) pure tone sweep centered on the test frequency at 20 dB above threshold, sampling every 1/25-oct. Neural signals were amplified and filtered (2400A; Dagan, Minneapolis, MN, USA), passed through a spike signal enhancer (40-46-1; FHC, Bowdoinham, ME), and digitized for analysis (DAP5016a; Microstar Laboratories).

A frequency response area was measured using tone bursts at various intensities throughout the mouse's audible range (4-60 kHz). From these data, the CF of a small cluster of units was determined using MATLAB (MathWorks, Natick, MA, USA). At this point, the neuronal tracer(s) was deposited iontophoretically using a high voltage, constant current source (CS 3; Midgard/Stoelting) set at 5 μA and 50% duty cycle for 5-10 min. After a rest period of 5 min the electrode was withdrawn, the craniotomy covered with bone wax, and the mouse returned to its cage. A survival period of 10-18 days ensured adequate filling of cells by the neuronal tracers.

Eleven successful iontophoretic injections were recovered from the mouse inferior colliculus after determining the best frequency of the site using the multi-unit tuning curve maker. Ten of the injections yielded a laminar distribution of the tracer as determined by a reconstruction of the reaction product through serial sections of the IC and illustrated by a photograph through a representative section of each case. They are presented in ascending order with respect to frequency at the injection site. A color-coded illustration of the injection sites is shown along with its corresponding IC outline (bottom right).

Supplementary Figure 1. Photomicrographs through the middle of the injection sites in coronal sections of the mouse IC. When reconstructed in 3D, each injection site formed a layer approximately 50-100 μm thick. These are combined in the drawing at the bottom right illustrating the tonotopic organization. *Abbreviations*: inferior colliculus (IC); kilohertz (kHz). Scale bar = 250 μm.

Supplementary Material 2 – Cell body size data – Results

Supplementary Figure 2. Plots of cell body sizes for major neurons of the LSO in male and female mice. **A.** The mean and standard deviation for the cell body silhouette area were calculated for principal and intrinsic neurons and for male and female mice. Overall, there is no difference in principal (*p=0.9998*) or intrinsic (*p=0.9707*) cell body size between the sexes. **B.** Likewise, there are no size changes with respect to age (from 3-8 months). **C.** The data represent four classes identified by their projections and cell body location: Principal, PO, intrinsic and shell neurons. A two-way ANOVA revealed significant differences between the principal, PO, intrinsic, and shell neurons. There are no size differences between the CV labeled neurons and the principal neurons ($F(105,438) = 0.86$, $p=0.8408$), and a slight but not statistically significant difference for the CV labeled neurons and the intrinsic efferents ($F(1,103) = 3.09$, $p=0.0816$). *Abbreviations:* Female (F); male (M); periolivary (PO).

Supplementary Table 1. Cell body silhouette area (µm^2) showing the similarity in principal and intrinsic cell size for mice aged between 3-8 months. Two-way ANOVA testing revealed no significant difference in cell size for neurons labeled in mice aged 3-8 months old for all cell types: ipsilateral and contralateral principal neurons, cholinergic neurons, and injected neurons ($F(1318,1287) = 0.99$, $p=0.5728$).

	**Principal Neurons**		**Intrinsic Neurons**	
	Ipsilateral	Contralateral	Cholinergic	Injected
**Number of cases**	4	4	7	5
**Ages (months)**	6, 7, 8	6, 7, 8	3, 4, 6, 8	4, 5, 6
**Number of cells**	911	947	1121	647
**Median area (µm^2)**	131.5	127.0	84.67	106.8
**Mean (µm^2) ± St. Dev**	134.5 ± 38.59	132.3 ± 36.89	97.92 ± 36.40	109.4 ± 33.78

Supplementary Table 2. Cell body silhouette area (µm^2) showing the similarity in principal and intrinsic cell size for male and female mice. Two-way ANOVA testing revealed no significant difference in cell size between principal neurons labeled in female and male mice ($F(1,713) = 0.09$, $p=0.7702$) and between intrinsic neurons labeled in either sex ($F(1,443) = 0.67$, $p=0.4127$).

	**Female**		**Male**	
Neurons:	Principal	Intrinsic	Principal	Intrinsic
**Number of cases**	5	5	5	5
**Number of cells**	915	745	1170	802
**Median area (µm^2)**	123.4	88.41	126.7	96.79
**Mean (µm^2) ± St. Dev**	126.5 ± 37.41	100.7 ± 36.40	128.0 ± 37.19	103.1 ± 33.69

Supplementary Table 3. Cell body silhouette area (μm²) for the LSO neuronal cohorts stained by different methods. A parametric T-test revealed no statistically significant difference between the ipsilateral and contralateral principal LSO neurons (*p=0.4126*). Two-way ANOVA compared the three methods of labeling the intrinsic neurons and revealed no significant difference ($F(61,84) = 0.59$, *p=0.9845*).

	**Principal Neurons**		**Intrinsic Neurons**		
	Ipsilateral	Contralateral	ChAT	AChE	Injected
**Number of cases**	4	4	4	3	5
**Number of cells**	679	611	369	243	430
**Median (μm²)**	124.4	126.5	83.77	84.75	107.1
**Mean (μm²) ± St. Dev**	129.3 ± 37.37	131.2 ± 36.87	94.02 ± 33.44	96.23 ± 38.22	110.9 ± 33.38

Supplementary Material 3—Somato-dendritic alignment

Labeled principal and intrinsic neurons were photographed and drawn in coronal sections cut at 60 μm thickness. The angle between the long axis of the principal cell body and dendritic vector was computed in the coronal plane from 12 cases. The average angle between the long axis and dendritic vector for the primary dendrites was relatively small, indicating alignment for these two neuronal features (principal neuron = 7.24 ± 10.42˚; intrinsic neuron = 6.49 ± 9.33˚; Supplementary Table 4).

We performed a similar analysis on 71 previously published LSO principal cells from different species stained by the Golgi method or Lucifer Yellow and presented in the coronal plane (cat: Cant, 1984; Helfert and Schwartz, 1986, gerbil: Helfert and Schwartz, 1987; Sanes et al., 1990, mouse: Ollo and Schwartz, 1979, rat: Rietzel and Friauf, 1998, and human: Kulesza, 2007). These cells exhibited extensive labeling of their dendrites and their average combined angle was 13.31 ± 11.32˚. *Welch's t test* (*p=0.0783)* confirmed no significant difference between somatic long axis and dendritic vector orientation for LSO cells with extensive dendritic staining. These data show that the somatic long axis is a reliable indicator its dendritic orientation.

Supplementary Figure 3. Cell body long axis and dendritic vector analysis in the LSO revealed no significant difference when comparing angle orientation of the two neuronal features. A. Drawing illustrates alignment of somatic long axis (black line) and dendritic vectors (red arrow) of principal neurons (gray) in a middle LSO section. **B.** A higher magnification of the ventromedial area outlined in A, illustrating the frequent alignment of soma and dendrites. **C.** Drawing of somatic long axis and dendritic vectors of intrinsic efferent neurons in the middle LSO section. **D.** A higher magnification of the boxed area in C, showing frequent alignment between soma and dendrites. *Key*: Angles of 30°, 60° and 90° degrees are included for reference. Note the general alignment between cell orientation and its dendritic

vector. Scale bar equals 100 µm (A, C) and 50 µm (B, D). **E.** Plot of the angle (˚) differences between soma long axes and dendritic vectors of labeled principal and intrinsic efferent neurons. The angles between the long axis and dendrite vector of each individual principal (red), intrinsic efferent (black) and Golgi and intracellularly labeled principal neurons from the literature (blue) were calculated. The points on the plot represent the angle difference for each neuron analysed. Golgi and intracellular labeling of principal neurons from the literature of cat, gerbil, mouse, rat and human LSO principal cells were included. These data illustrate that somatic orientation infers the overall dendritic orientation.

Supplementary Table 4. Angle measurements between cell body long axis and corresponding dendritic vector of labeled LSO principal and intrinsic neurons. Small degree values indicate close alignment between cell body long axis and dendritic vectors.

	Principal	Intrinsic
**Number of cases**	3	3
**Number of cells**	298	384
**Median** (degrees°)	3.00	3.00
**Mean** ± **SD** (degrees°)	7.24 ± 10.42	6.49 ± 9.33

Supplementary Material 4 – Tonotopic Axis

Principal and intrinsic neurons exhibited an aligned somato-dendritic orientation (Supplementary Material 3). We have demonstrated that these cells are also aligned within their respective isofrequency lamina and to the tonotopic axis (principal neurons = 32.99 ± 24.55˚; intrinsic neuron = 29.70 ± 22.51˚). These two cell types were not significantly different with respect to their relationship to the tonotopic axis across all sections (*Welch's t test:* rostral, *p=0.0809*; middle, *p=0.2610*; caudal, *p=0.1595;* combined total, *p=0.0118*). Despite the difference in their ascending and descending projections, principal and intrinsic neurons of the LSO exhibit similar structural alignment properties that would facilitate their frequency selectivity.

The PO neurons and shell efferents, which reside on the borders and immediately outside the LSO, exhibited somato-dendritic morphology that did not contribute to the structural laminae (middle, PO mean = 61.28 ± 24.62˚; shell efferent mean = 59.71 ± 24.39˚). PO neurons and shell efferents had on average similar angles of intersection with each other (*Welch's t tests:* rostral, *p=0.9992*; middle, *p=0.8094*; caudal, *p=0.9904*). These measurements are also consistent with the more orthogonal arrangement to the tonotopic axis of the LSO (Supplementary Table 5).

Neuronal alignment with respect to the tonotopic axis was performed on a series of LSO sections with principal neurons. The labeled cells created a columnar profile that defined an "isofrequency" sheet (Figure 6B,C) that ran the length of the LSO and hinged near the dorsal hilus (DH). An isofrequency sheet for each case was laid out on the 50th

percentile of the nucleus. A centroid was calculated for each isofrequency sheet (FIJI) and a (black) line drawn through the centroids of each LSO to connect the different sheets (Figure 6D). The connecting line represented the frequency axis of the nucleus and long axis of the sheet passed through the centroid at right angles (Figure 6D). We therefore used the tonotopic axis line produced by the algorithm for analysis of fiber and somatic-dendritic orientation.

The Hamilton-Jacobi Skeleton algorithm (He et al., 2021), which bisects a complex structure by following the curvature of the opposing borders, closely matched the frequency axis defined by us (black line, Figure 7D, *Welch's t test (p=0.2967)*). The spatial representation of different isofrequency sheets did not reveal any augmented representation that might imply functional specialization.

There was an obvious difference between the orientation of principal and intrinsic neurons and that of periolivary and shell neurons. On the basis of the angles of interaction with the frequency axis, these neurons were not aligned by with the frequency contours of the nucleus.

Supplementary Figure 4. Method process for quantifying neuronal alignment with the tonotopic axis. A series of LSO sections with labeled LSO neurons from an IC injections, cochlear injections, or cholinergic staining were traced, analysed for somatic orientation and quantified against a tonotopic axis derived from the Hamilton Jacobi algorithm. A single representative section is shown. The first step for this analysis was to trace the labeled cells and LSO border (top row, left). The traced neurons were put through a FIJI script to show the output of the somatic long-axis for each cell represented by a blue line (top row, right). The shape of the LSO nucleus was processed as a binary image (second row, left) into the Hamilton Jacobi algorithm to produce a skeletal output of the LSO nucleus shape to represent a tonotopic axis (red line, second row, right). The FIJI output of the somatic long-axes lines (blue) was superimposed onto the red tonotopic axis line derived from the algorithm (third row, left). A grey dashed line was extended from the blue somatic long-axis line to intersect with the tonotopic axis so the angles could be readily calculated as seen in the inset region (third row, right). *Inset region*: examples for how the angle (blue) for each somatic long-axis line (grey) was calculated against the perpendicular intersection at that point along the tonotopic axis (green, 90˚) and converted as an absolute value. A smaller angle difference inferred that the cell was orientated perpendicular to the tonotopic axis.

Supplementary Material 5 – Figure 5. Comparison of anatomically derived isofrequency layers (left) and electrophysiologically derived isofrequency lines (right). The anatomical laminae represent a collection of reconstructed profiles of chromogenically-recovered injection sites at their midpoint from CBA/CaH mice. Profiles are color coded with respect to the frequency spectrum (lower left). The isofrequency lines were taken from Figure 7B (Stiebler and Ehret, 1985) where they connected points of equivalent frequency as recorded in the IC of the house mouse. We color coded their map to the frequency spectrum. The tonotopic organization for the IC is remarkably consistent given the different methods, histology, mouse strains, and eras. kHz, kilohertz. The image approval for our adaptation of Stiebler and Ehret (1985) from John Wiley and Sons via the Copyright Clearance Centre; license number 5402831110296.

Supplementary Table 5. Descriptive statistics of LSO neuron subclasses and their alignment to the perpendicular intersection with the tonotopic axis. Principal and intrinsic neurons have greater alignment to the perpendicular intersection of the tonotopic axis than the periolivary and shell neurons. Angle deviations below 45° indicate parallel alignment to the perpendicular intersection, and angle deviations above 45° indicate perpendicular alignment. *Abbreviations*: Principal neurons (P); Periolivary neurons (PO); Intrinsic neurons (I); Shell neurons (S). Regardless of the position in the LSO, the principal and intrinsic neurons are aligned: Rostral – *p=0.0824*; Middle – *p=0.0694*; Caudal – *p=0.1643*; Total (combined across all sections) – *p = 0.0612*.

	**Rostral**				**Middle**				**Caudal**			
	P	PO	I	S	P	PO	I	S	P	PO	I	S
**Number of cases**	5	5	5	5	5	5	5	5	5	5	5	5
**Number of cells**	121	11	113	7	367	29	379	21	181	23	170	19
**Median** (degrees°)	43.00	68.00	33.00	71.00	25.00	73.00	23.00	71.00	30.00	73.00	30.00	74.00
**Mean ± SD** (degrees°)	40.25 ± 23.58	64.18 ± 17.90	35.10 ± 24.98	64.14 ± 13.04	30.56 ± 25.04	61.28 ± 24.62	27.95 ± 22.10	59.71 ± 24.39	33.06 ± 23.31	68.22 ± 16.26	30.03 ± 21.20	67.32 ± 20.16
