# Supplementary figures and images for: The lateral superior olive in the mouse: Two systems of projecting neurons
Source: Front Neural Circuits. 2022 Oct 20;16:1038500. doi: 10.3389/fncir.2022.1038500 (PMC9630946; doi:10.3389/fncir.2022.1038500)

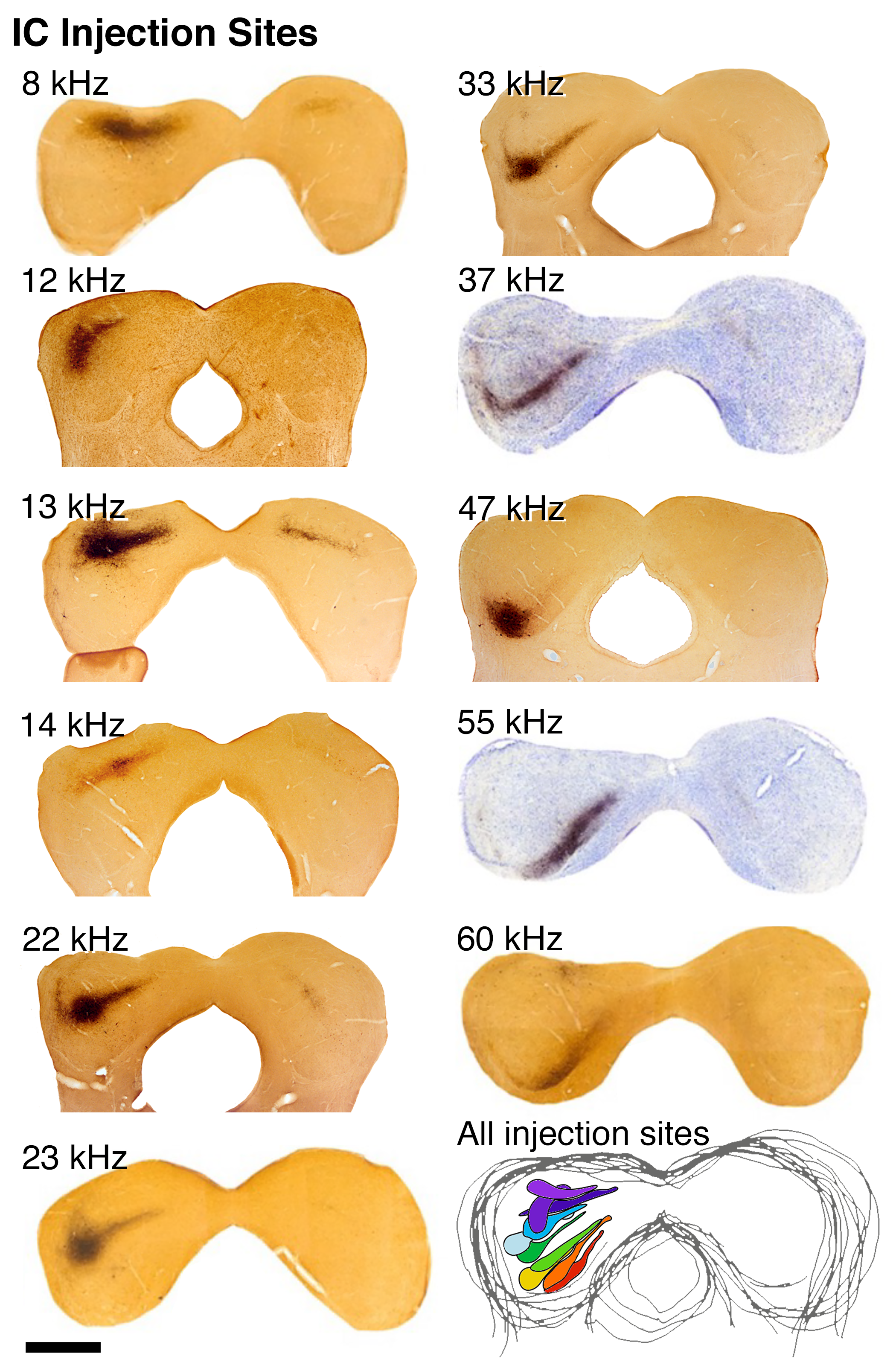

Supplement: Supplementary file 2 [file Image_1.TIF]

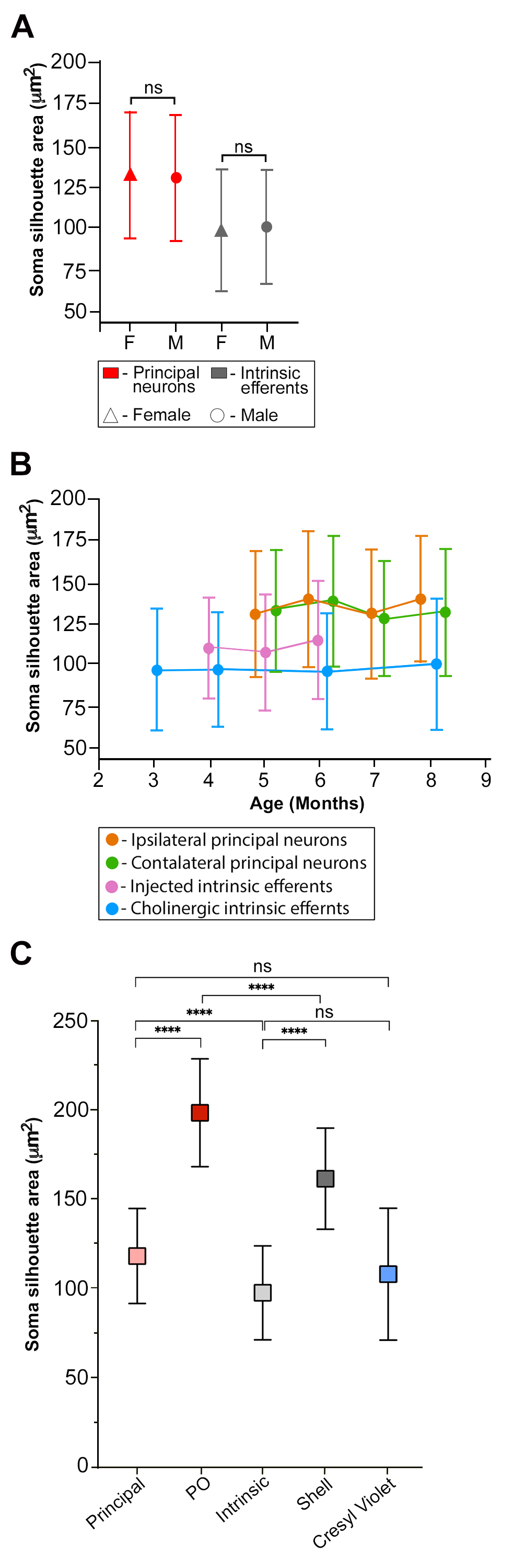

Supplement: Supplementary file 3 [file Image_2.TIF]

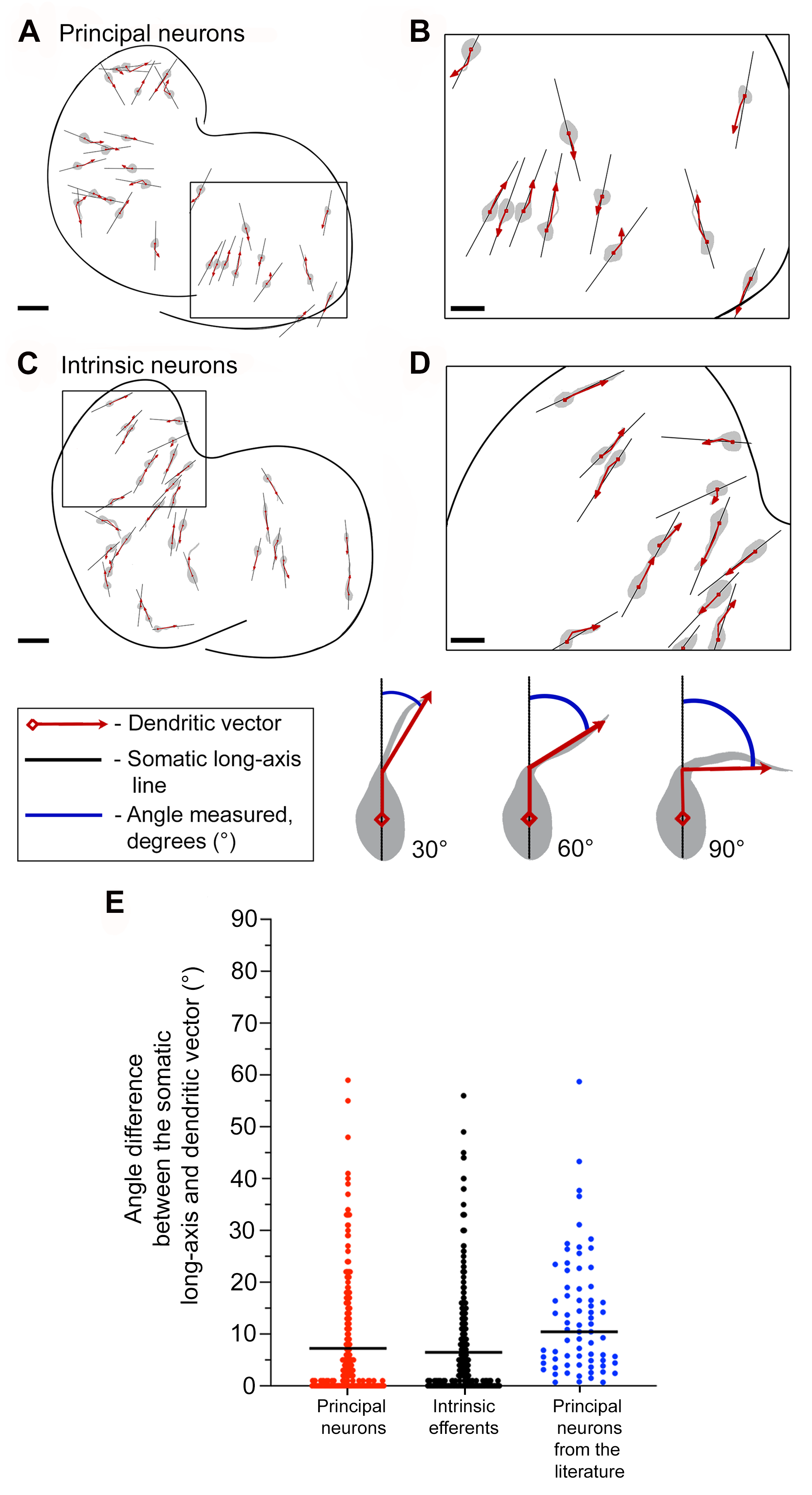

Supplement: Supplementary file 4 [file Image_3.TIF]

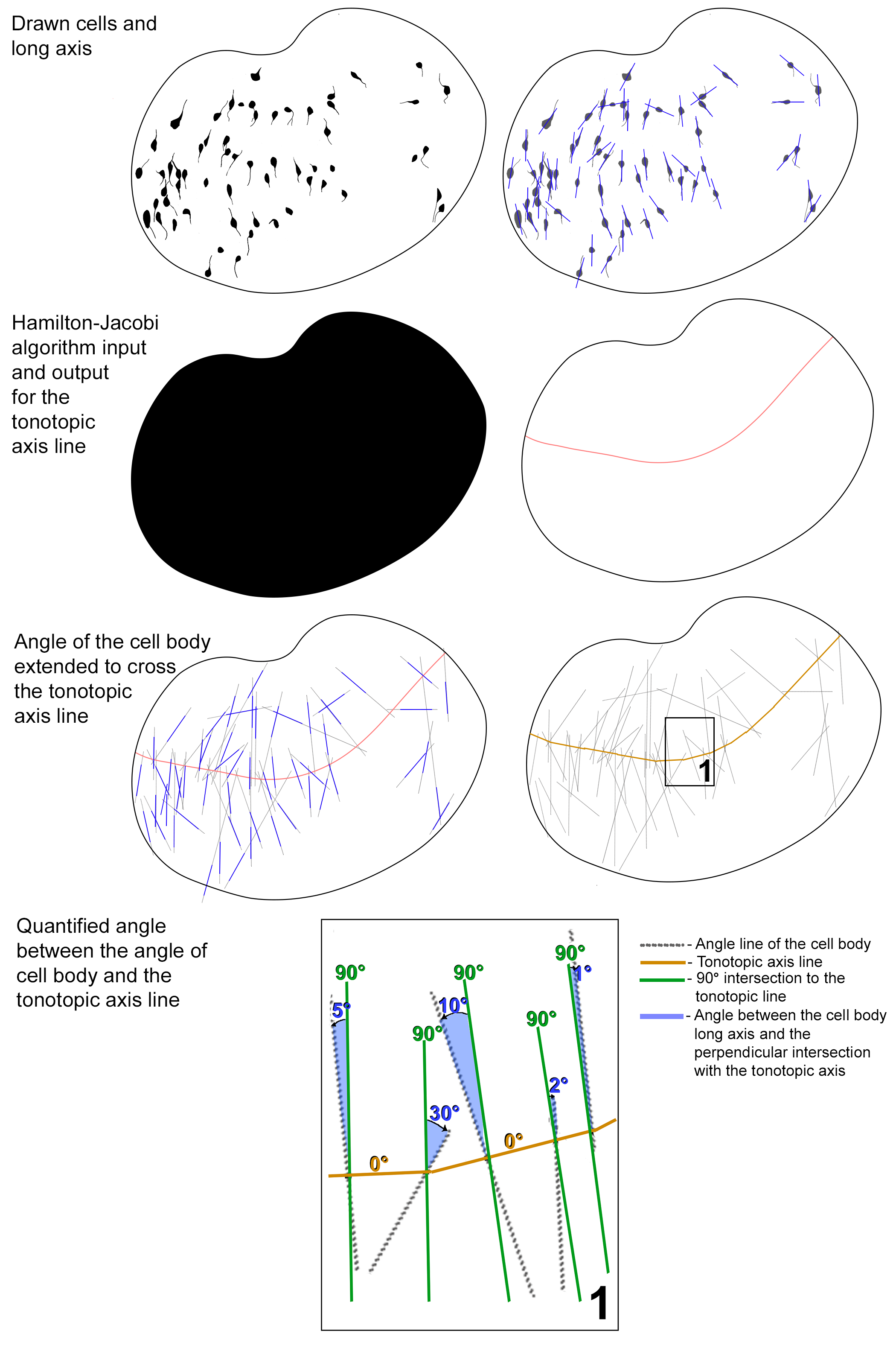

Supplement: Supplementary file 5 [file Image_4.TIF]

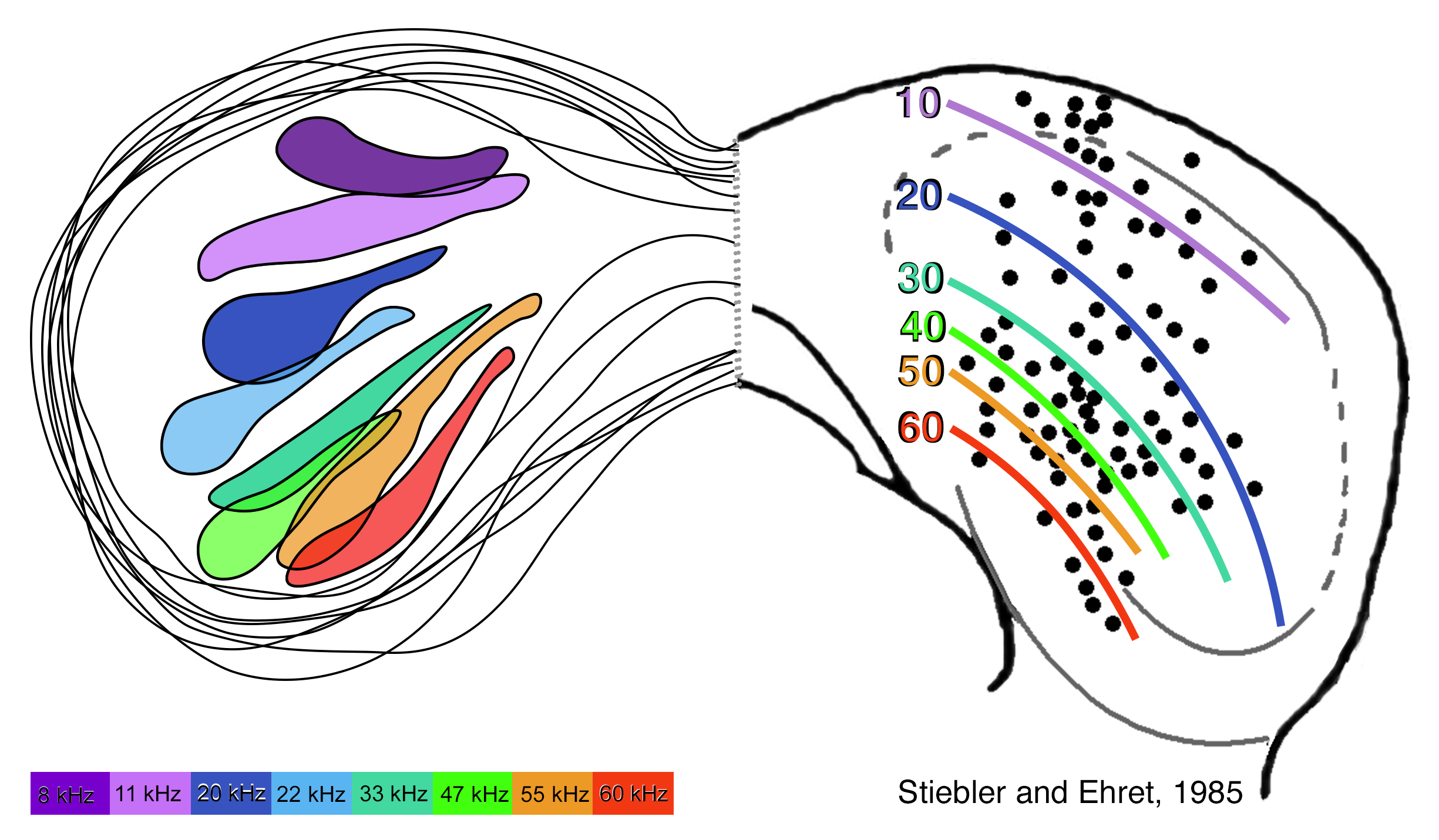

Supplement: Supplementary file 6 [file Image_5.TIF]
